# *CONSTANS* Polymorphism Modulates Flowering Time and Maturity in Soybean

**DOI:** 10.3389/fpls.2022.817544

**Published:** 2022-03-17

**Authors:** Mohammad Abdul Awal Khan, Shouwei Zhang, Reza Mohammad Emon, Fulu Chen, Wenwen Song, Tingting Wu, Shan Yuan, Cunxiang Wu, Wensheng Hou, Shi Sun, Yongfu Fu, Bingjun Jiang, Tianfu Han

**Affiliations:** ^1^MARA Key Laboratory of Soybean Biology (Beijing), Institute of Crop Sciences, Chinese Academy of Agricultural Sciences, Beijing, China; ^2^Plant Breeding Division, Bangladesh Institute of Nuclear Agriculture, Mymensingh, Bangladesh

**Keywords:** soybean, *GmCOL* orthologue, natural variation, flowering time, maturity group

## Abstract

*CONSTANS* (*CO*) plays a critical role in the photoperiodic flowering pathway. However, the function of soybean *CO* orthologs and the molecular mechanisms in regulating flowering remain largely unknown. This study characterized the natural variations in *CO* family genes and their association with flowering time and maturity in soybeans. A total of 21 soybean *CO* family genes (*GmCOL*s) were cloned and sequenced in 128 varieties covering 14 known maturity groups (MG 0000-MG X from earliest to latest maturity). Regarding the whole genomic region involving these genes, *GmCOL1*, *GmCOL3*, *GmCOL8*, *GmCOL9*, *GmCOL10*, and *GmCOL13* were conserved, and the remaining 15 genes showed genetic variation that was brought about by mutation, namely, all single-nucleotide polymorphisms (SNPs) and insertions-deletions (InDels). In addition, a few genes showed some strong linkage disequilibrium. Point mutations were found in 15 *GmCOL* genes, which can lead to changes in the potential protein structure. Early flowering and maturation were related to eight genes (*GmCOL1*/*3*/*4*/*8*/*13*/*15*/*16*/*19*). For flowering and maturation, 11 genes (*GmCOL2*/*5*/*6*/*14*/*20*/*22*/*23*/*24*/*25*/*26*/*28*) expressed divergent physiognomy. Haplotype analysis indicated that the haplotypes of *GmCOL5-Hap2*, *GmCOL13-Hap2/3*, and *GmCOL28-Hap2* were associated with flowering dates and soybean maturity. This study helps address the role of *GmCOL* family genes in adapting to diverse environments, particularly when it is necessary to regulate soybean flowering dates and maturity.

## Introduction

Plants can adapt to different environmental conditions in response to various day lengths (photoperiods). In the photoperiodic flowering pathway, *CONSTANS* (*CO*), which is a B-box-containing transcription factor (Robson et al., 2001; Gangappa and Botto, 2014), plays a key role (Putterill et al., 1995; Suarez-Lopez et al., 2001). CO also possesses a CONSTANS, CONSTANS-LIKE, TIMING OF CAB1 (CCT) domain at its C-terminus involved in DNA binding (Strayer et al., 2000; Robson et al., 2001). CYCLING DOF FACTOR (CDF) family proteins bind to the *CO* promoter to repress its transcription in the morning (Imaizumi et al., 2005; Fornara et al., 2009). FLAVIN-BINDING, KELCH REPEAT, and F-BOX1 (FKF1) interact with GIGANTEA (GI) to degrade the CDF1 protein, which results in the elevation of *CO* mRNA (Sawa et al., 2007). CO acts in the phloem of leaf vascular tissues to activate *FLOWERING LOCUS T* (*FT*) expression, which causes flowering under linkage disequilibrium (LD) conditions (Takada and Goto, 2003; An et al., 2004). SUPPRESSOR OF PHYTOCHROME A*-*105 (SPA) proteins interact with the CCT domain of CO (Laubinger et al., 2006). CO is ubiquitinated by CONSTITUTIVE PHOTOMORPHOGENIC 1 (COP1) and degraded by the 26S proteasome (Jang et al., 2008; Liu L. J. et al., 2008). HIGH EXPRESSION OF OSMOTICALLY RESPONSIVE GENES1 (HOS1) interacts with CO and participates in the degradation of CO mediated by red light (Lazaro et al., 2012, 2015). Nucleoporin96 (Nup96) interacts with HOS1 to gate CO protein levels (Cheng et al., 2020). CO protein is stable under the light in the evening but degraded in the morning or in the dark under LD conditions (Valverde et al., 2004). All of the abovementioned comments are based on studies in *Arabidopsis*.

As a short-day plant (SDP) distributed over a vast range of latitudes, soybean is characterized as having 14 maturity groups from MG 0000 to MG X (Liu et al., 2017; Jiang et al., 2019). There are hundreds of genetic loci associated with flowering time and maturity in soybean, among which *E1* (Xia et al., 2012), *E2* (Watanabe et al., 2011), *E3* (Watanabe et al., 2009), *E4* (Liu B. et al., 2008), *E6* (Fang C. et al., 2021), *E9* (Kong et al., 2014), *E10* (Samanfar et al., 2017), *E11* (Wang et al., 2019), *J* (Lu et al., 2017; Yue et al., 2017) and *Tof11/GmPRR37/GmPRR3b* (Li et al., 2020; Lu et al., 2020; Wang et al., 2020), *GmTof16* (Dong et al., 2021b), *GmFUL* (Dong et al., 2021a; Sun et al., 2021; Yue et al., 2021), and *GmLUX* (Bu et al., 2021; Fang X. et al., 2021) are molecularly identified. There are 28 *CO* orthologs in soybean (Fan et al., 2014), but the functions of most of the CO orthologs remain uncharacterized. It has been shown that *GmCOL1a* and *GmCOL1b* can fully complement the late-flowering phenotype of *the co1* mutant in *Arabidopsis* (Wu et al., 2014). In contrast, *GmCOL1a* and *GmCOL1b* repressed flowering in soybean under long-day conditions (Cao et al., 2015; Wu et al., 2019).

Natural variation is associated with photoperiodic flowering and adaptation in different species (Alonso-Blanco et al., 2005; Balasubramanian et al., 2006; Rosas et al., 2014; Li et al., 2017; Lu et al., 2017; Bao et al., 2019; Jiang et al., 2019; Wu et al., 2020). In this study, 128 soybean varieties were selected and planted, covering all 14 maturity groups from MG 0000 to MG X with a continuous distribution in maturity groups (Jiang et al., 2019). Due to the short duration of the project, 21 of the 28 soybean *COL* genes (Fan et al., 2014) were sequenced, and their sequence polymorphisms were analyzed. Furthermore, we studied the haplotypes of these soybean *GmCOL* genes to discover their natural variations in association with flowering time and maturity. These results suggested that some natural variations in 21 soybean *GmCOL* genes were associated with flowering date and maturity.

## Materials and Methods

### Plant Materials

Soybean varieties covering all 14 maturity groups (MG 0000-MG X) were assessed in this study (Jiang et al., 2019). In this study, 64 Chinese and Russian soybean varieties and 64 North American maturity group standard varieties were included, covering MG 0000-MG X, for a total of 128 accessions analyzed (Table 1).

**TABLE 1:** Soyabean varieties and maturity groups.

MGR (maturity group reference) varieties		

Maturity group	North American varieties	Chinese-Russian varieties
MG 0000		Star4/75, Hujiao07-2479, Hujiao07-2123, Dongnong 36, Paula, R-4, Dongnong 41, Lingbei 8
MG 000	Maple Presto, OAC Vision, Rassvet, Jug 30, Mageva	R-2, Heihe 35
MG 00	Canatto, Maple Ridge, Daksoy, McCall, Agassiz	Mengdou 32, Beidou 16, Dongnong 44, Mengdou 11
MG 0	Traill, Chico, Barnes, Norpro, Dawson	Jiangmodou 1, Heihe 18, Heihe 43, Heihe 27, Beidou 37, Dengke 1, Fengshou 12, Dongnong 4, Hefeng 25
MG I	Haroson, Kato, Parker, Granite, NE1900	Heinong 16, Taixingheidou, Heinong 26, Suinong 14
MG II	Holt, Olympus, Century 84, IL1, LN92-7369	Jilin 20, Yongchengzihuadou, Xiangchundou 24, Tiefeng 19
MG III	Athow, LN89-5699, KS3494, IL2, Williams 82	Zhonghuang 13, Zhonghuang 30, Tiefeng 33, Zhongdou 39, Xudou 9, Tiefeng 31, Jindou 19, Huachun 6, Huaidou 9
MG IV	Flyer, Omaha, Calhoun, CF461, UA-4805	Zheng 92116, Guandou 2, Jindou 39, Shanning 16, Houzimao
MG V	Nathan, Hollady, Hutcheson, R01-3474F, TN04-5321	Shangdou 14, Dian 86-4, Diandou 7
MG VI	Desha, Musen, D95-6271, G01-PR16, Boggs	Zhongdou 38, Wuhuasiyuehuang, Suxiandou 19, Nannong 493-1
MG VII	Stonewall, Benning, Santee, Hagood	Huangfengwo, Tongshanbopihuang, Hengyangbayueqing, Nanxiadou 25
MG VIII	Motte, Dowling, Crockett, Prichard, IAC-8, CIGRAS-51, CIGRAS-06	Aijiaoqing, Pingguohuangdou, Nandou 12, Shangraodaqingsi, Lanxidaqingdou, Qiudou 1, Nandou 17, Jiangledaqingdou, Guixia 3
MG IX	Jupiter, Alamo, FT-15, UFV-3	
MG X	I.C. 192	Zigongdongdou

### Investigation of Flowering and Maturity Dates

The soybeans were planted in soil in 10-L pots and grown under natural conditions in Haidian District, Beijing (39.95°N, 116.32°E) on May 27, 2015, and May 18, 2016 (Jiang et al., 2019). After emergence (VE), seedlings of similar size were selected so that each pot contained five uniform plants. Each variety was planted in three pots. The four developmental stages of soybean, namely, VE, R1, R7, and R8 (Fehr and Caviness, 1977), were investigated, and the average value of three pots for each variety was used for statistical analysis.

### DNA Isolation, PCR, and Sequencing

Genomic DNA was extracted from fresh trifoliolate leaves using the standard cetyltrimethylammonium bromide (CTAB) method (Jiang et al., 2019). To amplify the 21 soybean *GmCOL* genes, 36 PCR primer pairs were used. *GmCOL9*/*15*/*16*/*20*/*28* was amplified with 2 primer pairs: *GmCOL4* with 11 primer pairs and *GmCOL1*/*2*/*3*/*5*/*6*/*8*/*10*/*13*/*14*/*19*/*22*/*23*/*24*/*25*/*26* with 1 primer pair. The sequences of these primers and the template designation are listed in Supplementary Table 1. Target regions were amplified with the high-fidelity polymerase of KOD-Plus-Neo and KOD-FX. Their reaction conditions were 94°C for 2 min (98°C for 10 s, 68°C for 3 min), 35 cycles, and 68°C for 10 min. The PCR products were directly sequenced using the Sanger method (TSINGKE Biological Technology Company, BGI and Omega Genetics, Beijing, China). The polymorphic site information of the 21 soybean *GmCOL* family genes is listed in Supplementary Table 2.

### Data Mining and Sequence Analysis

The annotated soybean *COL* gene sequence was downloaded from the Joint Genome Institute (JGI) Phytozome website^1^. The protein sequences of the annotated *Arabidopsis* genes (TAIR9 release) were downloaded from the Arabidopsis Information Resource (TAIR) website^2^. The *CO*-like gene sequence data were also collected from the USDA-ARS Soybean Genetics and Genomics Database (SoyBase Database^3^). The reference sequence of the soybean variety Williams 82 was obtained from the Phytozome (version 12.0) database^4^ as a reference design for the amplification and sequencing primers of the *COL* genes.

Sequencing was performed using an ABI3730 sequencer. Multiple sequence alignment, editing, and stitching were carried out using ClustalW in MEGA5 with default parameters. Single-nucleotide polymorphism (SNP) analysis was carried out using TASSEL5 software. The haplotype analysis of the sequencing results was conducted by DNAsp to determine whether the protein coding was affected. LD was also evaluated using TASSEL5 software. The statistical analysis was performed using “R” software.

## Results

### Diversity of Soybean Varieties in Flowering Time and Maturity

**FIGURE 1:**
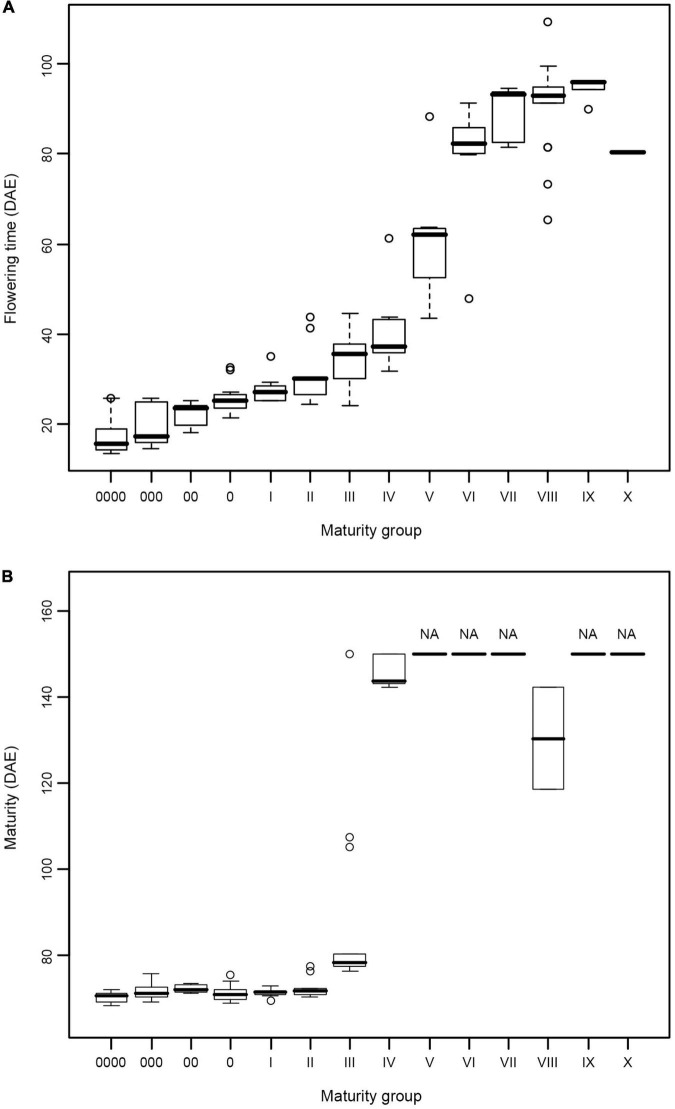
**(A,B)** Flowering time and maturity of soybean varieties from different maturity groups. Flowering time and maturity data were collected in 2016 from 128 soybean varieties covering 14 maturity groups. DAE, days after emergence.

The results of the growth traits are presented in Table 2. The varieties utilized in this experiment exhibited significant diversity in flowering time and maturity (Table 2). In 2015, 19 varieties, namely, MC119, MC63, MC69, MC70, MC54, MC121, MC122, MC123, MC72, MC124, MC125, MC126, MC48, MC64, MC65, MC66, MC67, MC68, and MC71, flowered but failed to reach R7. In 2016, 50 varieties did not reach R7 (Table 2). In 2015, the days to R1 from emergence (VE) ranged from 21 (MC82, MG 0) to 121 days (MC72, MG VIII) with a span of 100 days. In 2016, the span time was 96 days between MG 0000 (MC05, 13 days) and MG VIII (MC129, 109 days). In 2016, the days to R7 ranged from 68 (MC05 and MC74, MG 0000) to 144 days (MC42, MG IV), and the days to R8 ranged from 76 (MC05, MG 0000) to 109 days (MC37, MG III). The combination of maturity group information for the days of flowering time from the emergence and the days of beginning maturity from emergence in 2016 is plotted in a box diagram (Figure 1).

**TABLE 2:** Phenotypic variation of soybean varieties at Beijing.

Code	Variety name	MG	Phenotype in 2016		
			
			VE-R1	VE-R7	VE-R8
MC01	Star 4/75	0000	15.4 ± 1.2	70.6 ± 1.3	80.6 ± 0.61
MC02	Hujiao 07-2479	0000	16.4 ± 0.82	72.06 ± 1.27	81.26 ± 0.45
MC03	Hujiao 07-2123	0000	15.6 ± 0.50	71.13 ± 0.35	80.8 ± 0.41
MC04	Dongnong 36	0000	14.13 ± 0.51	69.13 ± 0.51	79.66 ± 0.97
MC05	Paula	0000	13.66 ± 0.48	68.4 ± 0.82	76.66 ± 0.48
MC06	R-4	0000	25.86 ± 0.74	71.46 ± 0.91	82.6 ± 0.82
MC07	Dongnong 41	0000	14.33 ± 0.48	69.46 ± 0.51	79.53 ± 0.91
MC74	Lingbei 8	0000	18.9 ± 1.0	68.4 ± 1.2	80.2 ± 1.5
MC75	Dongnong 41-C	0000	25.7 ± 1.4	70.5 ± 0.9	80.9 ± 1.0
MC08	Maple-Presto	000	15.53 ± 0.51	69.2 ± 1.01	77.66 ± 0.97
MC09	OAC-Vision	000	16.73 ± 0.79	69.8 ± 1.52	77.53 ± 0.91
MC10	Rassvet	000	17.33 ± 0.48	71.53 ± 0.91	79.8 ± 0.41
MC11	Jug-30	000	24.33 ± 0.48	71.2 ± 0.41	79.8 ± 0.67
MC12	Mageva	000	25.73 ± 0.45	75.66 ± 0.97	83.8 ± 0.86
MC73	R2	000	14.6 ± 0.9	70.6 ± 1.4	80.8 ± 2.1
MC76	Heihe 35	000	25.4 ± 1.5	73.5 ± o.5	87.5 ± 0.5
MC13	Canatto	00	18.2 ± 0.4	72.8 ± 1.8	85.2 ± 0.4
MC14	Maple-Ridge	00	18.4 ± 0.5	72.0 ± 1.0	84.7 ± 0.4
MC15	Daksoy	00	24.3 ± 0.4	71.3 ± 0.4	80.8 ± 0.3
MC16	McCall	00	23.6 ± 0.8	73.4 ± 1.2	85.8 ± 1.5
MC17	Agassiz	00	25.2 ± 0.4	71.6 ± 0.8	81.2 ± 0.4
MC77	Mengdou 32	00	22.6 ± 2.4	73.2 ± 1.0	86.3 ± 0.9
MC78	Beidou 16	00	19.9 ± 0.7	73.4 ± 1.5	85.4 ± 0.5
MC80	Dongnong 44	00	24.8 ± 1.0	71.3 ± 1.4	81.3 ± 0.9
MC81	Mengdou 11	00	24.3 ± 0.4	71.2 ± 1.3	81.6 ± 1.5
MC26	Traill	0	27.3 ± 0.4	73.3 ± 0.9	82.26 ± 0.7
MC27	Chico	0	26.4 ± 0.9	70.8 ± 1.5	81.4 ± 1.5
MC28	Barnes	0	26.5 ± 0.7	68.8 ± 1.3	78.4 ± 0.5
MC29	Norpro	0	25.3 ± 0.9	71.4 ± 0.50	82.1 ± 1.2
MC30	Dawson	0	23.6 ± 0.4	70.2 ± 1.37	80.4 ± 1.2
MC31	Jiangmodou 1	0	23.4 ± 0.9	69.7 ± 1.1	80.5 ± 0.9
MC82	Heihe 18	0	25.2 ± 1.5	72.0 ± 1.0	80.8 ± 1.3
MC83	Heihe 43	0	26.4 ± 0.5	71.9 ± 1.6	82.6 ± 0.7
MC84	Heihe 27	0	23.6 ± 0.4	70.8 ± 1.0	81.6 ± 0.5
MC85	Beidou 37	0	21.5 ± 0.9	69.5 ± 0.9	80.2 ± 1.2
MC86	Dengke1	0	23.8 ± 0.4	69.6 ± 0.4	80.4 ± 0.5
MC97	Fengshou 12	0	24.6 ± 0.9	71.0 ± 1.0	80.8 ± 1.0
MC88	Dongnong-4	0	32.2 ± 1.0	74.1 ± 0.8	85.8 ± 0.6
MC89	Hefeng 25	0	32.7 ± 0.4	75.5 ± 0.9	85.7 ± 0.7
MC23	Haroson	I	25.2 ± 0.45	71.2 ± 0.4	82.2 ± 0.4
MC24	Kato	I	27.5 ± 0.9	72.2 ± 0.4	81.5 ± 0.9
MC25	Parker	I	25.4 ± 0.9	71.4 ± 0.9	80.3 ± 0.9
MC26	Granite	I	25.3 ± 0.9	71.3 ± 0.9	78.4 ± 0.9
MC27	NE1900	I	35.2 ± 0.4	70.7 ± 1.0	80.1 ± 0.3
MC90	Heinong 16	I	28.5 ± 0.9	71.6 ± 0.9	79.4 ± 0.5
MC91	Taixingheidou	I	26.6 ± 0.9	72.73 ± 1.6	79.3 ± 1.7
MC92	Heinong 26	I	29.4 ± 0.5	70.8 ± 0.6	78.8 ± 0.9
MC93	Suinong 14	I	27.2 ± 1.5	69.4 ± 1.3	78.2 ± 1.3
MC28	Holt	II	30.3 ± 2.4	70.8 ± 0.8	81.8 ± 1.5
MC29	Olympus	II	27.7 ± 0.9	72.3 ± 0.4	82.0 ± 1.0
MC30	Century-84	II	26.6 ± 0.4	71.6 ± 0.8	81.0 ± 1.2
MC31	IL1	II	30.4 ± 5.5	70.2 ± 1.7	79.6 ± 2.5
MC32	LN92-7369	II	30.4 ± 1.4	76.2 ± 1.0	107.9 ± 1.6
MC94	Jilin 20	II	25.2 ± 1.0	70.3 ± 0.9	78.2 ± 1.0
MC95	Yongchengzihuadou	II	41.3 ± 1.2	72.1 ± 1.2	87.7 ± 1.2
MC96	Xiangchundou 24	II	43.8 ± 1.0	77.4 ± 0.5	86.6 ± 1.2
MC98	Tiefeng 19	II	24.4 ± 0.5	71.2 ± 1.2	81.2 ± 1.7
MC33	Athow	III	30.3 ± 0.9	76.3 ± 0.9	106.3 ± 0.9
MC34	Zhonghuang 13	III	43.3 ± 0.4	78.4 ± 0.9	106.4 ± 0.9
MC35	Zhonghuang 30	III	37.8 ± 0.9	78.3 ± 0.9	105.5 ± 1.4
MC36	LN89-5699	III	32.3 ± 0.9	80.2 ± 1.5	108.2 ± 0.4
MC37	KS3494	III	37.4 ± 0.5	79.8 ± 0.9	109.4 ± 1.5
MC38	IL2	III	42.1 ± 0.8	105.2 ± 2.3	105.3 ± 5.1
MC39	Williams 82	III	36.8 ± 0.3	107.3 ± 0.8	108.1 ± 2.3
MC97	Tiefeng 33	III	29.5 ± 0.5	78.7 ± 0.4	106.8 ± 1.2
MC99	Zhongdou 39	III	44.5 ± 0.5	77.4 ± 0.5	105.2 ± 1.8
MC100	Xudou 9	III	37.9 ± 0.7	78.0 ± 1.0	106.4 ± 1.5
MC101	Tiefeng 31	III	24.2 ± 1.1	77.2 ± 1.3	105.2 ± 1.1
MC102	Jindou 19	III	25.8 ± 1.3	77.4 ± 1.5	104.8 ± 1.5
MC103	Huachun 6	III	31.4 ± 0.5	77.3 ± 0.4	105.6 ± 0.9
MC104	Huaidou 9	III	34.3 ± 0.4	ND	ND
MC40	Flyer	IV	35.5 ± 1.4	143.0 ± 1.4	ND
MC41	Omaha	IV	38.0 ± 1.0	143.4 ± 1.4	ND
MC42	Calhoun	IV	37.3 ± 0.4	144.4 ± 0.5	ND
MC43	CF461	IV	31.8 ± 1.0	143.7 ± 0.4	ND
MC44	UA-4805	IV	61.2 ± 0.4	ND	ND
MC105	Zheng 92116	IV	37.2 ± 0.4	ND	ND
MC106	Guandou 2	IV	35.8 ± 1.0	ND	ND
MC107	Jindou 39	IV	36.2 ± 1.3	142.9 ± 1.0	ND
MC108	Shanning 16	IV	43.4 ± 0.8	142.3 ± 0.4	ND
MC109	Houzimao	IV	43.7 ± 1.4	143.3 ± 1.1	ND
MC45	Nathan	V	60.2 ± 1.0	ND	ND
MC46	Holladay	V	62.2 ± 1.5	ND	ND
MC47	Hutcheson	V	63.2 ± 1.0	ND	ND
MC48	R01-3474F	V	62.2 ± 1.5	ND	ND
MC49	TN04-5321	V	63.6 ± 1.5	ND	ND
MC110	Shangdou 14	V	44.7 ± 0.4	ND	ND
MC111	Dian 86-4	V	43.6 ± 1.5	ND	ND
MC113	Diandou 7	V	88.3 ± 0.9	ND	ND
MC50	Desha	VI	48.0 ± 0.8	ND	ND
MC51	Musen	VI	80.0 ± 1.0	ND	ND
MC52	D95-6271	VI	82.3 ± 1.5	ND	ND
MC53	G01-PR16	VI	81.6 ± 0.9	ND	ND
MC54	Boggs	VI	79.8 ± 1.5	ND	ND
MC112	Zhongdou 38	VI	83.6 ± 2.5	ND	ND
MC114	Wuhuasiyuehuang	VI	86.3 ± 0.9	ND	ND
MC108	Suxiandou 19	VI	85.8 ± 1.2	ND	ND
MC116	Nannong 493-1	VI	91.2 ± 0.4	ND	ND
MC55	Stonewall	VII	81.4 ± 0.5	ND	ND
MC57	Santee	VII	82.6 ± 1.8	ND	ND
MC58	Hagood	VII	94.6 ± 1.2	ND	ND
MC56	Benning	VII	93.3 ± 0.9	ND	ND
MC117	Hunagfengwu	VII	93.7 ± 0.4	ND	ND
MC118	Tongshanbopihuang	VII	93.2 ± 1.2	ND	ND
MC60	Motte	VIII	93.3 ± 0.9	ND	ND
MC61	Dowling	VIII	95.8 ± 1.5	ND	ND
MC62	Crockett	VIII	65.4 ± 0.2	142.2 ± 0.5	158.0 ± 1.0
MC128	Hengyangbayueqing	VII	96.9 ± 1.0	ND	ND
MC119	Nanxiadou 25	VII	92.6 ± 0.9	ND	ND
MC63	Prichard	VIII	94.8 ± 1.0	ND	ND
MC69	CIGRAS-51	VIII	81.4 ± 0.5	ND	ND
MC70	CIGRAS-06	VIII	81.5 ± 0.9	ND	ND
MC54	Aijiaoqing	VIII	94.3 ± 0.4	ND	ND
MC121	Pingguohuangdou	VIII	92.9 ± 1.0	ND	ND
MC122	Nandou 12	VIII	91.3 ± 0.4	ND	ND
MC123	Shangraodaqingsi	VIII	92.8 ± 1.0	ND	ND
MC72	Zigongdongdou	VIII	73.4 ± 1.5	ND	ND
MC124	Lanxidaqingdou	VIII	91.6 ± 0.5	ND	ND
MC125	Qiudou 1	VIII	94.3 ± 0.4	ND	ND
MC126	Nandou 17	VIII	91.6 ± 0.4	ND	ND
MC48	Jiangledaqingdou	VIII	99.5 ± 0.9	ND	ND
MC129	Guixia 3	VIII	109.2 ± 1.0	118.4 ± 0.2	ND
MC64	Jupiter	IX	96.3 ± 0.9	ND	ND
MC65	Alamo	IX	96.0 ± 1.3	ND	ND
MC66	FT-15	IX	94.2 ± 1.3	ND	ND
MC67	UFV-3	IX	96.2 ± 1.0	ND	ND
MC68	IAC-8	IX	90.0 ± 1.0	ND	ND
MC71	I.C.-192	X	80.4 ± 1.5	ND	ND

*ND, Not available data; VE, Variation after emergence.*

### *GmCOL* Gene Sequencing and Mutation Study

**FIGURE 2:**
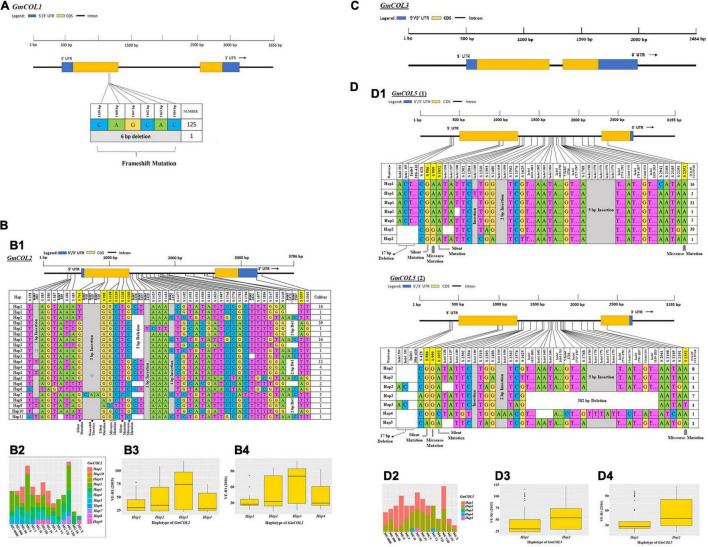
Haplotype analysis, distribution in different maturity groups, and flowering time (VE-R1) in the main haplotypes of approximately four *GmCOL* family genes. **(A)** Haplotype of *GmCOL1*. **(B,B1)** Haplotypes of *GmCOL2*. The haplotype is shown as a linear combination of alleles. The site combination labeled with the yellow outline represents the tagging haplotype (the composition of the haplotype depends on fewer and critical variation sites). **(B2)** Haplotype distribution of *GmCOL2* in different maturity groups. **(B3)** Flowering time (VE-R1) of the main haplotypes of *GmCOL2* in 2015. **(B4)** Flowering time (VE-R1) of the main haplotypes of *GmCOL2* in 2016. **(C)** Gene structure of *GmCOL3*. **(D,D1)** Haplotypes of *GmCOL5* (1) and *GmCOL5* (2). **(D2)** Haplotype distribution of *GmCOL5* in different maturity groups. **(D3)** Flowering time (VE-R1) of the main haplotypes of *GmCOL5* in 2015. **(D4)** Flowering time (VE-R1) of the main haplotypes of *GmCOL5* in 2016.

A large number of mutated sites were found in *GmCOL2*, *GmCOL5*, *GmCOL14*, *GmCOL16*, *GmCOL20*, and *GmCOL28* (Figures 2B1,D1 and Supplementary Figures 1G1,I1, 2B1,H1). A majority of the mutation sites were in gene intron regions, but a few were located in protein-coding regions caused by insertions-deletions (InDels) and substitutions. In addition, *GmCOL6*, *GmCOL22*, *GmCOL23*, *GmCOL24*, *GmCOL25*, and *GmCOL26* showed high mutation frequencies in the protein-coding regions (Supplementary Figures 1, 2). In contrast, *GmCOL4*, *GmCOL15*, and *GmCOL19* expressed lower mutational occurrences (Supplementary Figures 1A1,H1, 2A1). The exception was *GmCOL3*, which did not show mutations (Figure 2C) in either the intron or exon region of the entire gene. *GmCOL3* is a highly conserved gene with no change in sequence. However, the amino acid sequences encoded by *GmCOL1*, *GmCOL8*, *GmCOL9*, *GmCOL10*, and *GmCOL13* were not affected (Figure 2A and Supplementary Figures 1C1-F1).

### Soybean *COL* Gene Family Exhibited Different Linkage Disequilibrium

**FIGURE 3:**
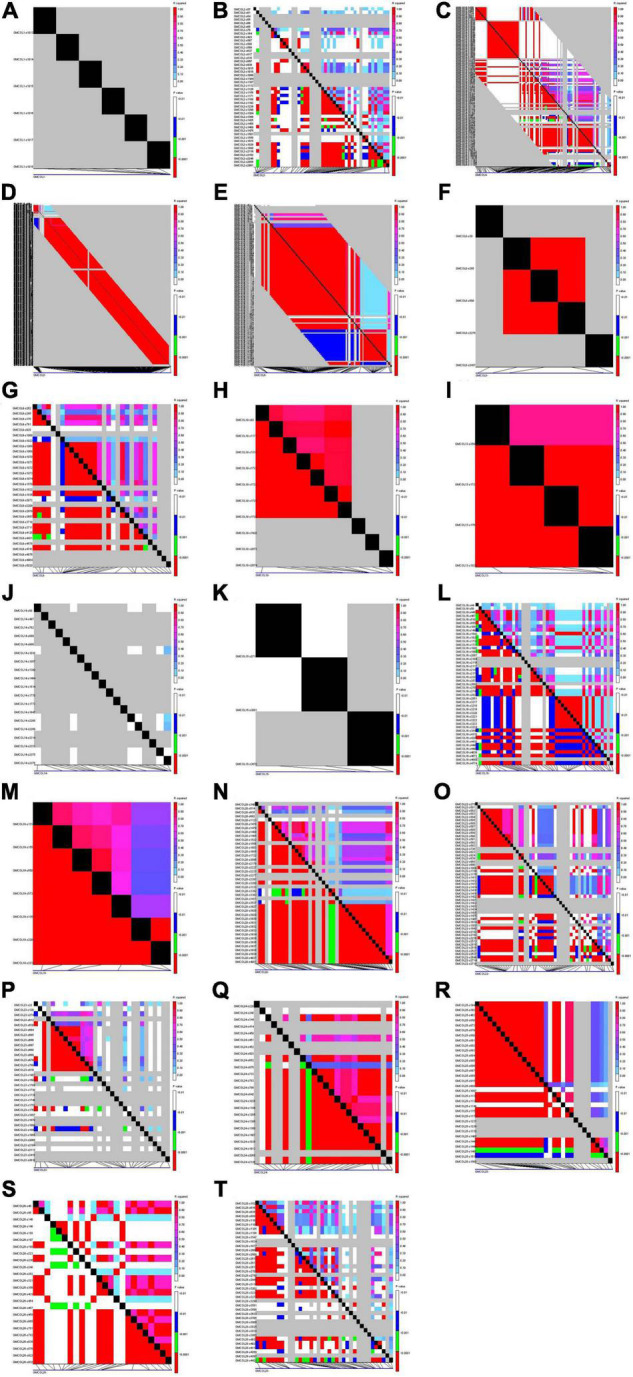
Linkage disequilibrium of *GmCOL* family genes in soybean. **(A)** Linkage disequilibrium (LD) of *GmCOL1*. **(B)** LD of *GmCOL2*. **(C)** LD of *GmCOL4*. **(D)** LD of *GmCOL5*. **(E)** LD of *GmCOL6*. **(F)** LD of *GmCOL8*. **(G)** LD of *GmCOL9*. **(H)** LD of *GmCOL10*. **(I)** LD of *GmCOL13*. **(J)** LD of *GmCOL14*. **(K)** LD of *GmCOL15*. **(L)** LD of *GmCOL16*. **(M)** LD of *GmCOL19*. **(N)** LD of *GmCOL20*. **(O)** LD of *GmCOL22*. **(P)** LD of *GmCOL23*. **(Q)** LD of *GmCOL24*. **(R)** LD of *GmCOL25*. **(S)** LD of *GmCOL26*. **(T)** LD of *GmCOL28*.

Linkage and association mapping were drawn among the SNPs of the gene *via* TASSEL5. *GmCOL5* across the region almost from the starting to the end site presented a strong LD by completing the haplotype block (Figure 3D). *GmCOL20*, *GmCOL24*, and *GmCOL25* across the region expressed strong LD as a haplotype block (Figures 3N,Q,R), and the polymorphic sites of *GmCOL6*, *GmCOL8*, *GmCOL10*, *GmCOL13*, and *GmCOL19* showed a strong LD, with almost the entire region being a haplotype block (Figures 3E,F,H,I,M). Although *GmCOL2*, *GmCOL16*, *GmCOL22*, *GmCOL26*, and *GmCOL28* had a high-sequence polymorphism, the entire region distributed a few LDs (Figures 3B,L,O,S,T) with the same high-sequence polymorphism of *GmCOL4*, *GmCOL9*, and *GmCOL23*, and quite a few strong LDs were distributed broadly (Figures 3C,G,P). *GmCOL1*, *GmCOL14*, and *GmCOL15* did not reveal any LD (Figures 3A,J,K). *GmCOL3* was the most conserved gene, showing no polymorphic site(s).

### Haplotype Analysis and Its Association With Maturity Groups of Soybean Varieties

Based on LD and sequence clustering, haplotype definition was analyzed for the 21 soybean *GmCOL* gene families in the 128 varieties investigated. A total of 21 haplotypes of the *GmCOL* gene family variation types and polymorphic sites used for composing haplotypes are listed in Figure 2 and Supplementary Tables 3, 4 (Supplementary Figures 1, 2), respectively. Supplementary Table 5 shows 21 *GmCOL* protein groups associated with haplotypes.

*GmCOL2-Hap2* was dominant in the 41 accessions covering all maturity groups, except MG X. *GmCOL2-Hap4* showed early flowering compared with the other haplotype groups (Figures 2B3,B4, Table 2, and Supplementary Table 3). *GmCOL4-Hap1* accounted for 99 varieties, with all maturity groups excluding MG X (Supplementary Figure 1A2). *GmCOL4-Hap1* had an earlier flowering date in 2016. *GmCOL5-Hap2* was present in 56 accessions, covering all maturity groups (Figure 2D1). *GmCOL6-Hap1* was the most abundant in 70 varieties from different maturity groups, including MG 0000-MG V and MG VIII-MG X (Supplementary Figure 1B1). Among the 11 haplotypes, *GmCOL9-Hap1* was dominant in 67 varieties from maturity groups MG 0000-MG X (Supplementary Figures 1D1,D2). *GmCOL9-Hap3* and *GmCOL9-Hap4* were distributed in a few varieties, and the flowering time for both haplotypes was early. *GmCOL10-Hap1* was the most abundantly expressed in 91 varieties from all maturity groups except MG X (Supplementary Figures 1E1,E2). *GmCOL16-Hap1* was rich in accessions distributed in the maturity groups MG 0000-MG VIII (Supplementary Figures 1I1,I2). *GmCOL20-Hap2* was present in 52 varieties from all maturity groups (MG 0000-MG X) (Supplementary Figures 2B1,B2). Among all the haplotypes of *GmCOL28*, *GmCOL28-Hap1* was the most common, accounting for 59 accessions, and was distributed in the maturity groups MG 0000, MG 00, MG 0, MG I-MG VIII, and MG X (Supplementary Figures 2H1,H2). In this experiment, no haplotype variants were found in *GmCOL1* and *GmCOL3* (Figures 2A,C). The *Hap1* series of haplotypes of *GmCOL8/13/14/15/19/22/23/24/25/26* was the most abundant in the varieties and was distributed in all 14 maturity groups (Supplementary Figures 1C1,F1,G1,H1, 2A1,C1,D1,E1,F1,G1).

### Haplotypes Associated With Flowering Time

The haplotype groups of 21 gene families are presented in Table 3. An analysis of variance (ANOVA) was performed to elucidate the natural variations in flowering time (VE-R1) of the *GmCOL* gene families based on haplotype groups. In this analysis, eight genes (*GmCOL2*/*5*/*9*/*13*/*15*/*16*/*25*/*28*) showed significant results from the first emergence to the first flowering date (VE-R1) in both years, and three genes [*GmCOL8* (2015), *GmCOL22* (2015), and *GmCOL26* (2016)] exhibited significant results in a single year (Supplementary Table 6). The remaining genes (*GmCOL4*/*6*/*10*/*14*/*19*/*20*/*23*/*24*) did not show significant results in these years. Notably, *GmCOL1* and *GmCOL3* were the most conserved, and no polymorphisms in the coding region were observed.

**TABLE 3:** Haplotype frequency of *GmCOL* family genes.

	Haplotype										
*GmCOL2*	*Hap1*	*Hap2*	*Hap3*	*Hap4*	*Hap5*	*Hap6*	*Hap7*	*Hap8*	*Hap9*	*Hap10*	*Hap11*
Accessions	18	41	20	17	3	3	3	3	1	1	1
*GmCOL4*	*Hap1*	*Hap2*	*Hap3*	*Hap4*	*Hap5*						
Accessions	99	7	4	4	1						
*GmCOL5*	*Hap1*	*Hap2*	*Hap3*	*Hap4*	*Hap5*						
Accessions	51	56	1	1	1						
*GmCOL6*	*Hap1*	*Hap2*	*Hap3*	*Hap4*	*Hap5*						
Accessions	70	46	5	1	1						
*GmCOL8*	*Hap1*	*Hap2*									
Accessions	88	11									
*GmCOL9*	*Hap1*	*Hap2*	*Hap3*	*Hap4*	*Hap5*	*Hap6*	*Hap7*	*Hap8*	*Hap9*	*Hap10*	*Hap11*
Accessions	67	25	4	4	3	1	1	1	1	1	1
*GmCOL10*	*Hap1*	*Hap2*	*Hap3*	*Hap4*							
Accessions	91	16	1	1							
*GmCOL13*	*Hap1*	*Hap2*	*Hap3*								
Accessions	122	5	1								
*GmCOL14*	*Hap1*	*Hap2*	*Hap3*								
Accessions	114	1	1								
*GmCOL15*	*Hap1*	*Hap2*	*Hap3*	*Hap4*							
Accessions	107	5	2	1							
*GmCOL16*	*Hap1*	*Hap2*	*Hap3*	*Hap4*	*Hap5*	*Hap6*	*Hap7*	*Hap8*	*Hap9*		
Accessions	54	25	24	6	5	4	2	2	1		
*GmCOL19*	*Hap1*	*Hap2*	*Hap3*	*Hap4*	*Hap5*						
Accessions	99	17	2	2	1						
*GmCOL20*	*Hap1*	*Hap2*	*Hap3*	*Hap4*	*Hap5*	*Hap6*	*Hap7*	*Hap8*	*Hap9*	*Hap10*	
Accessions	19	52	27	8	2	2	1	1	1	1	
*GmCOL22*	*Hap1*	*Hap2*	*Hap3*	*Hap4*	*Hap5*	*Hap6*					
Accessions	110	7	4	3	2	1					
*GmCOL23*	*Hap1*	*Hap2*	*Hap3*	*Hap4*	*Hap5*	*Hap6*	*Hap7*	*Hap8*	*Hap9*	*Hap10*	
Accessions	80	4	3	2	1	1	1	1	1	1	
*GmCOL24*	*Hap1*	*Hap2*	*Hap3*	*Hap4*	*Hap5*						
Accessions	88	5	3	1	1						
*GmCOL25*	*Hap1*	*Hap2*	*Hap3*	*Hap4*	*Hap5*						
Accessions	89	9	6	6	1						
*GmCOL26*	*Hap1*	*Hap2*	*Hap3*	*Hap4*							
Accessions	61	56	7	2							
*GmCOL28*	*Hap1*	*Hap2*	*Hap3*								
Accessions	59	29	22								

**FIGURE 4:**
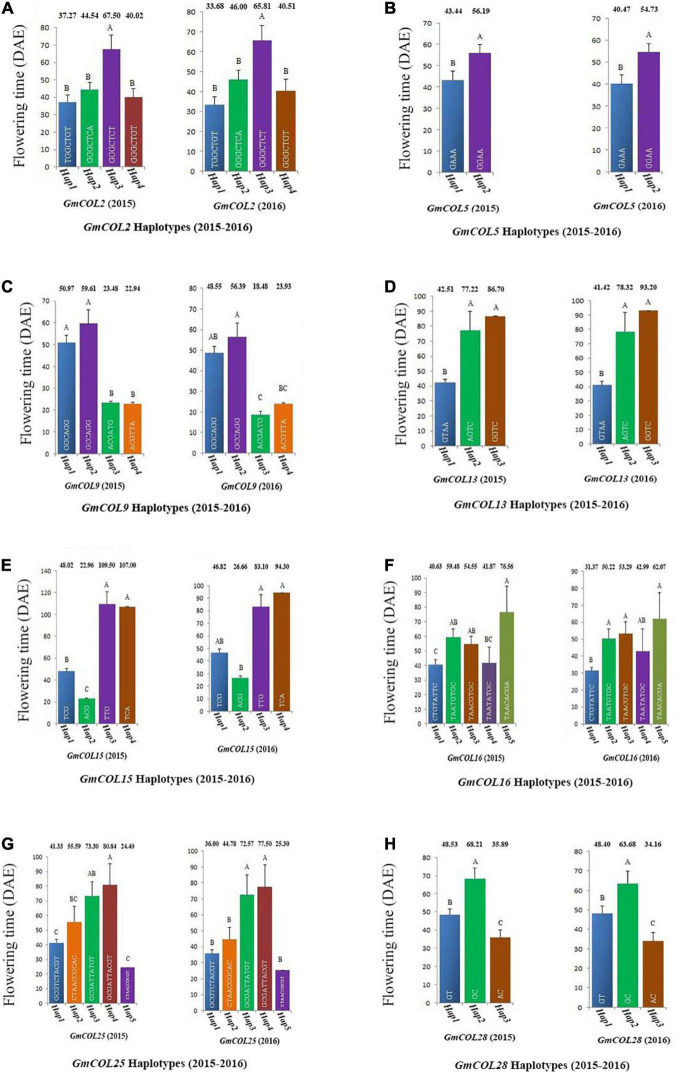
Haplotypes of *GmCOL* genes in relation to flowering time (2015–2016). The relationship between flowering time and haplotype of *GmCOL2* **(A)**, *GmCOL5* **(B)**, *GmCOL9* **(C)**, *GmCOL13* **(D)**, *GmCOL15* **(E)**, *GmCOL16* **(F)**, *GmCOL25* **(G)**, and *GmCOL28* **(H)**.

Among the 11 haplotype groups of *GmCOL2*, 4 were analyzed, and *GmCOL2-Hap3* was significantly different from every other haplotype group (Figure 4A). The *GmCOL2-Hap1*/*2*/*4* haplotype groups, which appeared in the above haplotype group, were not significantly different from each other. Analysis of *GmCOL5* showed that in both years two major haplotypes showed significant differences from each other, and *Hap2* was related to late flowering compared with *Hap1* (Figure 4B). In terms of *GmCOL5 Hap2* (S999), the original nucleotide (A^499^) was mutated to G^499^ in the coding sequence, resulting in a missense mutation in the amino acid sequence (K^167^ to E^167^). K^167^ was located between the B-box and the CCT domain. In comparison, *GmCOL9*, *Hap1*, and *Hap2* (Figure 4C) did not show significant differences from each other, and both were significantly different from *GmCOL9-Hap3* and *GmCOL9-Hap4* only in 2015. Likewise, *GmCOL13-Hap2*/*3* (Figure 4D) was closely associated with flowering time because both were significantly different from *GmCOL13-Hap1* in both years.

For *GmCOL13 Hap2/3* (S584), the original nucleotide (G^84^) was mutated to A^84^ in the coding sequence, resulting in a silent mutation in the no. 28 amino acid sequence, which was located in the first B-box. In both years, *GmCOL15-Hap3*/*4* (Figure 4E) showed a significant difference compared with *GmCOL15-Hap1*/*2*. For *GmCOL16*, the haplotypes showed non-significant results among the four haplotypes (*Hap1*, *Hap2*, *Hap3*, and *Hap4*) (Figure 4F). In the first year, five haplotypes of *GmCOL25* (*Hap1*, *Hap2*, *Hap3*, *Hap4*, and *Hap5*) were not significantly different from each other, whereas *Hap3* and *Hap4* were significantly different from *Hap1*, *Hap2*, and *Hap5* in the second year (Figure 4G). *Hap2* of *GmCOL28* was significantly different from *Hap1* and *Hap3* in both years (Figure 4H). For *GmCOL28 Hap2* (S1066), the original nucleotide (T^263^) was mutated to C^263^ in the coding sequence, resulting in a missense mutation in the amino acid sequence (V^88^ to A^88^), which was located in the first B-box, and may affect the protein and protein interaction. Thus, the results revealed that *Hap2 of GmCOL28* was closely associated with flowering time.

## Discussion

### Selected Soybean Varieties Showed Diversity in Flowering Time and Maturity

The varieties analyzed in this study showed the diversity of flowering time and maturity, which indicated that the flowering time (VE-R1) and the days from emergence to physiological maturity (VE-R7) were related to the variety trait and natural environment (Jiang et al., 2019). According to the photoperiod responses, many soybean varieties have evolved to adapt to a broad range of growing areas in China (Wang et al., 2016). The flowering time of the selected varieties ranged from 21 to 121 days and 13.66 to 109.2 days, and the maturity time ranged from 61.2 to 150.7 days and 68.4 to 144.4 days, respectively, for two consecutive years (2015 and 2016) (Jiang et al., 2019), indicating high diversity (Table 2). However, Paula showed the shortest VE-R1 in the 2nd year, with the maturity group MG 0000. Paula is recognized as a high-latitude cold region (HCR) soybean variety and HCR soybean, which typically matures early (Jia et al., 2014). This observation suggested that the maturity group MG 0000 was relatively stable for days to start flowering from emergence.

### *GmCOL* Orthologs Showed Divergence in Sequence Polymorphism

It has been shown that GmCOL1a (Glyma08g28370) and GmCOL1b (Glyma18g51320) in soybean are the closest *Arabidopsis* COL2 orthologs to CO (Thakare et al., 2010). The transgenic soybean line overexpressing *GmCOL1a* flowered late under long-day or natural conditions (Cao et al., 2015). There is a single-nucleotide substitution at the GmCOL1b CCT domain in the *gmcol1b* mutant, leading to the mutagenesis of conserved threonine at amino acid 314 into isoleucine, which results in early flowering of the *gmcol1b* mutant (Cao et al., 2015). In this study, the varieties that were taken for the observation of natural variations regarding flowering time and maturation showed divergent characteristics in flowering time (Jiang et al., 2019). Allelic variation can have an effect on flowering time (Irwin et al., 2016). In this study, the insertion and single-nucleotide substitution (InDel918, InDel919, InDel920, s958, s1138, s1139, s1218, and s1260) found in the first exon in *GmCOL2* led to missense mutations, which might have an effect on flowering time and maturity. The other genes in the *GmCOL* family showed various types of point mutations (Figure 2 and Supplementary Figures 1–5). The loss of functions may have an impact on gene function, and the presence of these mutations suggested that polymorphism could be the main cause of the diversity of soybean flowering time.

### Polymorphism of *GmCOL* Family Genes Was Associated With Flowering Time and Maturity

Haplotype-based analyses have been successfully carried out in different crops, such as maize (*Zea mays* L.) (Weber et al., 2009; Van Inghelandt et al., 2012; Lipka et al., 2013), rice (*Oryza sativa* L.) (Lestari et al., 2011; Yonemaru et al., 2012, 2014; Shao et al., 2013; Wu et al., 2020), and soybean (Choi et al., 2007; Li et al., 2009; Langewisch et al., 2014; Patil et al., 2016; Jiang et al., 2019). In this study, some polymorphic sites were identified and utilized by conducting a haplotype analysis of 21 soybean *GmCOL* gene families. Based on LD analysis and polymorphic sites, tagging haplotypes composed of some SNPs and InDels indicated that there were 7 polymorphic sites in *GmCOL2* and just 3 polymorphic sites in *GmCOL10* and 46 and 10 polymorphic sites in *GmCOL2* and *GmCOL10*, respectively (Supplementary Table 4). In addition, some haplotypes associated with flowering date and maturity (such as *GmCOL2-Hap1*, *Hap2*, and *Hap4*; *GmCOL9-Hap1*, *Hap2*, *Hap3*, and *Hap4*; *GmCOL13-Hap1*, *Hap2*, and *Hap3*; *GmCOL15-Hap1*, *Hap2*, *Hap3*, and *Hap4*; *GmCOL16-Hap1*, *Hap2*, *Hap3*, *Hap4*, and *Hap5*; and *GmCOL25-Hap1*, *Hap2*, *Hap3*, *Hap4*, and *Hap5*) were distributed in varieties with different maturity groups. The LD differences among the 21 soybean *COL* gene families may reflect selection pressure and, to some extent, the process of natural selection and domestication.

## Conclusion

In summary, 21 *GmCOL* genes exhibited natural divergence in association with flowering and growth periods. Significant changes were found in 21 *COL* genome sequences, among which *GmCOL1*, *GmCOL3*, *GmCOL8*, *GmCOL9*, *GmCOL10*, and *GmCOL13* were conserved, but the sequences of the remaining *COL* genes showed a wide range of changes that could alter their function. In this study, it was noticed that additional polymorphisms linked to the 21 *GmCOL* gene families in trait-controlling regions might have a significant impact on flowering time and maturity.

## Data Availability Statement

The datasets presented in this study can be found in online repositories. The names of the repository/repositories and accession number(s) can be found in the article/Supplementary Material.

## Author Contributions

MA conducted the major phenotypic and genotypic works. SZ, RE, WS, TW, and BJ joined the experiments. SY, CW, WH, SS, and YF provided the materials and technical supports. MA, RE, and BJ prepared the initial draft of the manuscript. FC rewrote the manuscript. FC, BJ, MA, RE, and TH revised the manuscript. TH coordinated and supervised the project. All authors read and approved the final manuscript.

## Conflict of Interest

The authors declare that the research was conducted in the absence of any commercial or financial relationships that could be construed as a potential conflict of interest.

## Publisher’s Note

All claims expressed in this article are solely those of the authors and do not necessarily represent those of their affiliated organizations, or those of the publisher, the editors and the reviewers. Any product that may be evaluated in this article, or claim that may be made by its manufacturer, is not guaranteed or endorsed by the publisher.

## Footnotes

1
https://phytozome-next.jgi.doe.gov/

2
www.arabidopsis.org

3
http://soybase.org/

4
http://phytozome.jgi.doe.gov/pz/portal.html

## Supplementary Material

Supplementary Figure 1 Haplotype analysis, distribution in different maturity groups, and flowering time (VE-R1) in the main haplotypes of approximately nine *GmCOL* family genes. **(A,A1)** Haplotypes of *GmCOL4*. **(A2)** Haplotype distribution of *GmCOL4* in different maturity groups. **(A3)** Flowering time (VE-R1) of the main haplotypes of *GmCOL4* in 2015. **(A4)** Flowering time (VE-R1) of the main haplotypes of *GmCOL4* in 2016. **(B,B1)** Haplotypes of *GmCOL6*. **(B2)** Haplotype distribution of *GmCOL6* in different maturity groups. **(B3)** Flowering time (VE-R1) of the main haplotypes of *GmCOL6* in 2015. **(B4)** Flowering time (VE-R1) of the main haplotypes of *GmCOL6* in 2016. **(C,C1)** Haplotypes of *GmCOL8*. **(C2)** Haplotype distribution of *GmCOL8* in different maturity groups. **(C3)** Flowering time (VE-R1) of the main haplotypes of *GmCOL8* in 2015. **(C4)** Flowering time (VE-R1) of the main haplotypes of *GmCOL8* in 2016. **(D,D1)** Haplotypes of *GmCOL9* (1) and *GmCOL9* (2). **(D2)** Haplotype distribution of *GmCOL9* in different maturity groups. **(D3)** Flowering time (VE-R1) of the main haplotypes of *GmCOL9* in 2015. **(D4)** Flowering time (VE-R1) of the main haplotypes of *GmCOL9* in 2016. **(E,E1)** Haplotypes of *GmCOL10*. **(E2)** Haplotype distribution of *GmCOL10* in different maturity groups. **(E3)** Flowering time (VE-R1) of the main haplotypes of *GmCOL10* in 2015. **(E4)** Flowering time (VE-R1) of the main haplotypes of *GmCOL10* in 2016. **(F,F1)** Haplotypes of *GmCOL13*. **(F2)** Haplotype distribution of *GmCOL13* in different maturity groups. **(F3)** Flowering time (VE-R1) of the main haplotypes of *GmCOL13* in 2015. **(F4)** Flowering time (VE-R1) of the main haplotypes of *GmCOL13* in 2016. **(G,G1)** Haplotypes of *GmCOL14*. **(G2)** Haplotype distribution of *GmCOL14* in different maturity groups. **(G3)** Flowering time (VE-R1) of the main haplotypes of *GmCOL14* in 2015. **(G4)** Flowering time (VE-R1) of the main haplotypes of *GmCOL14* in 2016. **(H,H1)** Haplotypes of *GmCOL15*. **(H2)** Haplotype distribution of *GmCOL15* in different maturity groups. **(H3)** Flowering time (VE-R1) of the main haplotypes of *GmCOL15* in 2015. **(H4)** Flowering time (VE-R1) of the main haplotypes of *GmCOL15* in 2016. **(I,I1)** Haplotypes of *GmCOL16* (1), *GmCOL16* (2), *GmCOL16* (3), and *GmCOL16* (4). **(I2)** Haplotype distribution of *GmCOL16* in different maturity groups. **(I3)** Flowering time (VE-R1) of the main haplotypes of *GmCOL16* in 2015. **(I4)** Flowering time (VE-R1) of the main haplotypes of *GmCOL16* in 2016.

Supplementary Figure 2 Haplotype analysis, distribution in different maturity groups, and flowering time (VE-R1) in the main haplotypes of approximately eight *GmCOL* family genes. **(A,A1)** Haplotypes of *GmCOL19*. **(A2)** Haplotype distribution of *GmCOL19* in different maturity groups. **(A3)** Flowering time (VE-R1) of the main haplotypes of *GmCOL19* in 2015. **(A4)** Flowering time (VE-R1) of the main haplotypes of *GmCOL19* in 2016. **(B,B1)** Haplotypes of *GmCOL20* (1), *GmCOL20* (2), and *GmCOL20* (3). **(B2)** Haplotype distribution of *GmCOL20* in different maturity groups. **(B3)** Flowering time (VE-R1) of the main haplotypes of *GmCOL20* in 2015. **(B4)** Flowering time (VE-R1) of the main haplotypes of *GmCOL20* in 2016. **(C,C1)** Haplotypes of *GmCOL22* (1) and *GmCOL22* (2). **(C2)** Haplotype distribution of *GmCOL22* in different maturity groups. **(C3)** Flowering time (VE-R1) of the main haplotypes of *GmCOL22* in 2015. **(C4)** Flowering time (VER1) of the main haplotypes of *GmCOL22* in 2016. **(D,D1)** Haplotypes of *GmCOL23* (1) and *GmCOL23* (2). **(D2)** Haplotype distribution of *GmCOL23* in different maturity groups. **(D3)** Flowering time (VE-R1) of the main haplotypes of *GmCOL23* in 2015. **(D4)** Flowering time (VE-R1) of the main haplotypes of *GmCOL23* in 2016. **(E,E1)** Haplotypes of *GmCOL24*. **(E2)** Haplotype distribution of *GmCOL24* in different maturity groups. **(E3)** Flowering time (VE-R1) of the main haplotypes of *GmCOL24* in 2015. **(E4)** Flowering time (VE-R1) of the main haplotypes of *GmCOL24* in 2016. **(F,F1)** Haplotypes of *GmCOL25*. **(F2)** Haplotype distribution of *GmCOL25* in different maturity groups. **(F3)** Flowering time (VE-R1) of the main haplotypes of *GmCOL25* in 2015. **(F4)** Flowering time (VE-R1) of the main haplotypes of *GmCOL25* in 2016. **(G,G1)** Haplotypes of *GmCOL26*. **(G2)** Haplotype distribution of *GmCOL26* in different maturity groups. **(G3)** Flowering time (VE-R1) of the main haplotypes of *GmCOL26* in 2015. **(G4)** Flowering time (VE-R1) of the main haplotypes of *GmCOL26* in 2016. **(H,H1)** Haplotypes of *GmCOL28* (1) and *GmCOL28* (2). **(H2)** Haplotype distribution of *GmCOL28* in different maturity groups. **(H3)** Flowering time (VE-R1) of the main haplotypes of *GmCOL28* in 2015. **(H4)** Flowering time (VE-R1) of the main haplotypes of *GmCOL28* in 2016.
