# Supplementary figures and images for: CONSTANS Polymorphism Modulates Flowering Time and Maturity in Soybean
Source: Front Plant Sci. 2022 Mar 17;13:817544. doi: 10.3389/fpls.2022.817544 (PMC8969907; doi:10.3389/fpls.2022.817544)

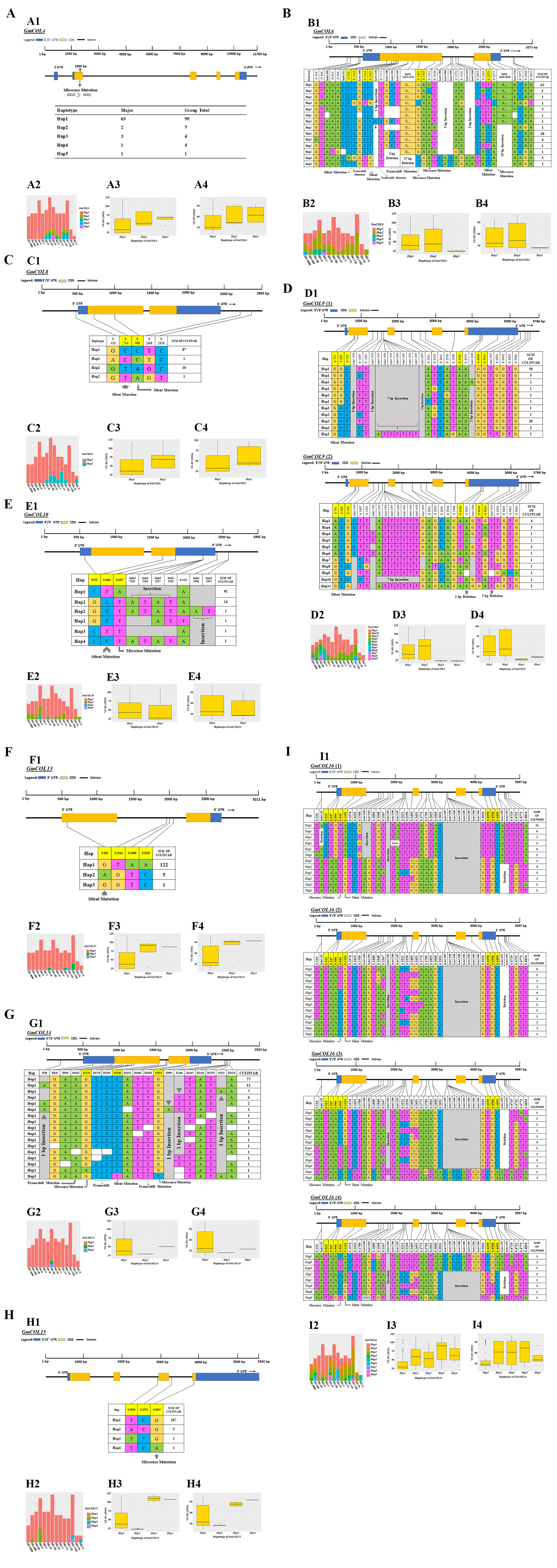

Supplement: Supplementary Figure 1 — Haplotype analysis, distribution in different maturity groups, and flowering time (VE-R1) in the main haplotypes of approximately nine GmCOL family genes. (A,A1) Haplotypes of GmCOL4. (A2) Haplotype distribution of GmCOL4 in different maturity groups. (A3) Flowering time (VE-R1) of the main haplotypes of GmCOL4 in 2015. (A4) Flowering time (VE-R1) of the main haplotypes of GmCOL4 in 2016. (B,B1) Haplotypes of GmCOL6. (B2) Haplotype distribution of GmCOL6 in different maturity groups. (B3) Flowering time (VE-R1) of the main haplotypes of GmCOL6 in 2015. (B4) Flowering time (VE-R1) of the main haplotypes of GmCOL6 in 2016. (C,C1) Haplotypes of GmCOL8. (C2) Haplotype distribution of GmCOL8 in different maturity groups. (C3) Flowering time (VE-R1) of the main haplotypes of GmCOL8 in 2015. (C4) Flowering time (VE-R1) of the main haplotypes of GmCOL8 in 2016. (D,D1) Haplotypes of GmCOL9 (1) and GmCOL9 (2). (D2) Haplotype distribution of GmCOL9 in different maturity groups. (D3) Flowering time (VE-R1) of the main haplotypes of GmCOL9 in 2015. (D4) Flowering time (VE-R1) of the main haplotypes of GmCOL9 in 2016. (E,E1) Haplotypes of GmCOL10. (E2) Haplotype distribution of GmCOL10 in different maturity groups. (E3) Flowering time (VE-R1) of the main haplotypes of GmCOL10 in 2015. (E4) Flowering time (VE-R1) of the main haplotypes of GmCOL10 in 2016. (F,F1) Haplotypes of GmCOL13. (F2) Haplotype distribution of GmCOL13 in different maturity groups. (F3) Flowering time (VE-R1) of the main haplotypes of GmCOL13 in 2015. (F4) Flowering time (VE-R1) of the main haplotypes of GmCOL13 in 2016. (G,G1) Haplotypes of GmCOL14. (G2) Haplotype distribution of GmCOL14 in different maturity groups. (G3) Flowering time (VE-R1) of the main haplotypes of GmCOL14 in 2015. (G4) Flowering time (VE-R1) of the main haplotypes of GmCOL14 in 2016. (H,H1) Haplotypes of GmCOL15. (H2) Haplotype distribution of GmCOL15 in different maturity groups. (H3) Flowering time (VE-R1) of the main haplotypes of Gm [file Image_1.JPEG]

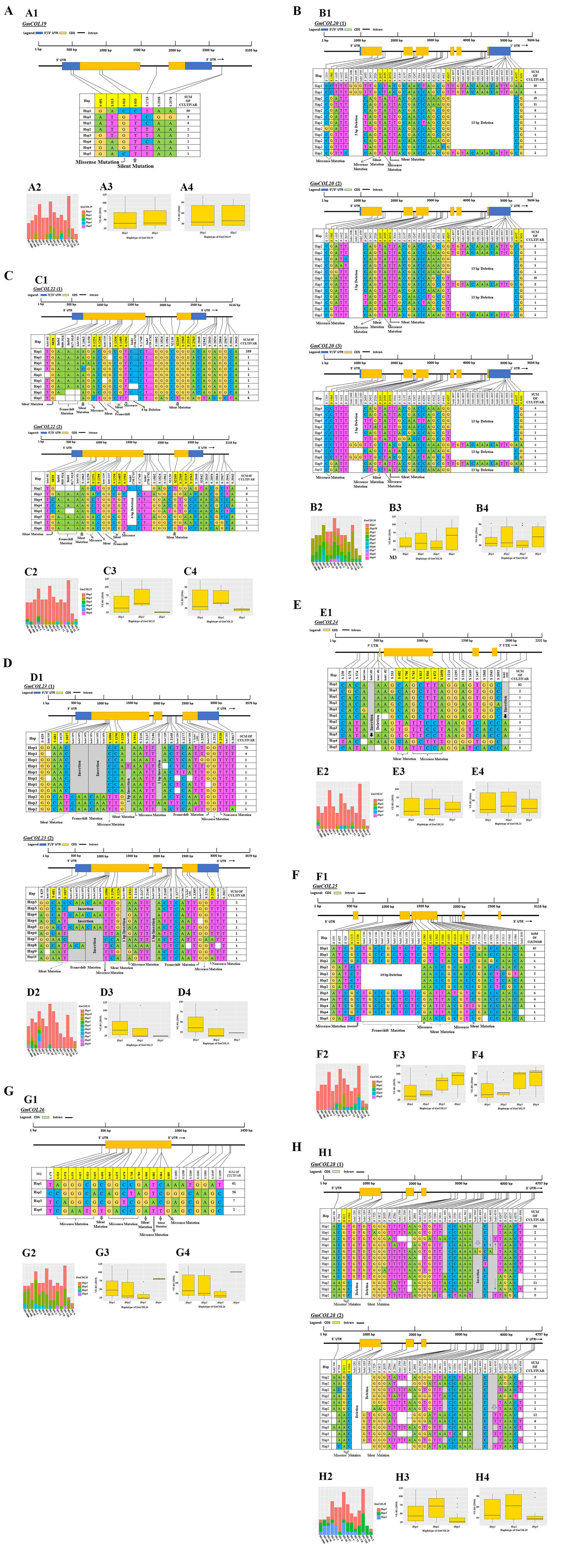

Supplement: Supplementary Figure 2 — Haplotype analysis, distribution in different maturity groups, and flowering time (VE-R1) in the main haplotypes of approximately eight GmCOL family genes. (A,A1) Haplotypes of GmCOL19. (A2) Haplotype distribution of GmCOL19 in different maturity groups. (A3) Flowering time (VE-R1) of the main haplotypes of GmCOL19 in 2015. (A4) Flowering time (VE-R1) of the main haplotypes of GmCOL19 in 2016. (B,B1) Haplotypes of GmCOL20 (1), GmCOL20 (2), and GmCOL20 (3). (B2) Haplotype distribution of GmCOL20 in different maturity groups. (B3) Flowering time (VE-R1) of the main haplotypes of GmCOL20 in 2015. (B4) Flowering time (VE-R1) of the main haplotypes of GmCOL20 in 2016. (C,C1) Haplotypes of GmCOL22 (1) and GmCOL22 (2). (C2) Haplotype distribution of GmCOL22 in different maturity groups. (C3) Flowering time (VE-R1) of the main haplotypes of GmCOL22 in 2015. (C4) Flowering time (VER1) of the main haplotypes of GmCOL22 in 2016. (D,D1) Haplotypes of GmCOL23 (1) and GmCOL23 (2). (D2) Haplotype distribution of GmCOL23 in different maturity groups. (D3) Flowering time (VE-R1) of the main haplotypes of GmCOL23 in 2015. (D4) Flowering time (VE-R1) of the main haplotypes of GmCOL23 in 2016. (E,E1) Haplotypes of GmCOL24. (E2) Haplotype distribution of GmCOL24 in different maturity groups. (E3) Flowering time (VE-R1) of the main haplotypes of GmCOL24 in 2015. (E4) Flowering time (VE-R1) of the main haplotypes of GmCOL24 in 2016. (F,F1) Haplotypes of GmCOL25. (F2) Haplotype distribution of GmCOL25 in different maturity groups. (F3) Flowering time (VE-R1) of the main haplotypes of GmCOL25 in 2015. (F4) Flowering time (VE-R1) of the main haplotypes of GmCOL25 in 2016. (G,G1) Haplotypes of GmCOL26. (G2) Haplotype distribution of GmCOL26 in different maturity groups. (G3) Flowering time (VE-R1) of the main haplotypes of GmCOL26 in 2015. (G4) Flowering time (VE-R1) of the main haplotypes of GmCOL26 in 2016. (H,H1) Haplotypes of GmCOL28 (1) and GmCOL28 (2). (H2) Haplotype distribution of GmC [file Image_2.JPEG]
