# Supplementary material for: CONSTANS Polymorphism Modulates Flowering Time and Maturity in Soybean
Source: Front Plant Sci. 2022 Mar 17;13:817544. doi: 10.3389/fpls.2022.817544 (PMC8969907; doi:10.3389/fpls.2022.817544)
Supplement: Supplementary file 3 [file Table_1.docx]

**Table S1** *GmCOL* genes amplification primer sequences

| **Gene** | **Template** | **Primer Name** | **Sequence (5’3’)** |
| --- | --- | --- | --- |
| *GmCOL1* | *GmCOL1* | *GmCOL*-1F | ACTCAAACTACAGTCAAGTTCAGTGTACC |
|  |  | *GmCOL*-1R | GGCCCAACGTACTTCATGTTAATCG |
| *GmCOL2* | *GmCOL2* | *GmCOL*-2F | GACAAAGGAAATGAGAGACTAGGGT |
|  |  | *GmCOL*-2R | TCAACCAAAACTCAAACAACAGTCA |
| *GmCOL3* | *GmCOL3* | *GmCOL*-3F | CCCCACTAGATAGACTTGGACTCACT |
|  |  | *GmCOL*-3R | GTCACTTGAAAGACAACCCAACACG |
| *GmCOL4* | *GmCOL4* | *GmCOL4*-1F | AAGTCCCAAGATCTGCAAGTA |
|  |  | *GmCOL4*-1R | CAAGGCAGCATCAATAAGATA |
|  |  | *GmCOL4*-2F | ACTGGCTGAGAGGATTCATAT |
|  |  | *GmCOL4*-2R | CGCTTCTCTTACTGTGTGTTG |
|  |  | *GmCOL4*-3F | GAGCAGGAGACATCTTGATCA |
|  |  | *GmCOL4*-3R | TGGCTGTAAACACTGTTCTTC |
|  |  | *GmCOL4*-4F | CTCTCTGTTCCCATTCTTCAT |
|  |  | *GmCOL4*-4R | GAAGCCATGAACACAAAATAC |
|  |  | *GmCOL4*-5F | GTTAGGTTCTGTTAGTTACAA |
|  |  | *GmCOL4*-5R | TATGGGGTAGGTTGTAATGTA |
|  |  | *GmCOL4*-6F | AACTCTTCTTCGATAAATTAC |
|  |  | *GmCOL4*-6R | TTTCAGCTTTGGACTTTATAG |
|  |  | *GmCOL4*-7F | TGTGGGGACCTTCTCATATTA |
|  |  | *GmCOL4*-7R | TTGGACCTGTTAATGGAATCA |
|  |  | *GmCOL4*-8F | CTATGCGACAAGAGTATGATG |
|  |  | *GmCOL4*-8R | GCAATTTTTTTAGCAGAGTAT |
|  |  | *GmCOL4*-9F | TTACCAAGCCCTTAGTTATTC |
|  |  | *GmCOL4*-9R | AAGTGAAAACCAATAAAAGTC |
|  |  | *GmCOL4*-10F | TCACATTTTTGTTTCTTACTA |
|  |  | *GmCOL4*-10R | TGCTTAATAGTGTTCTCATGA |
|  |  | *GmCOL4*-11F | TGTCCCGTGTAGGTTTAGAAG |
|  |  | *GmCOL4*-11R | TCTGAATGGAGTCAAAAGTTA |
| *GmCOL5* | *GmCOL5* | *GmCOL5*-1F | CATCTGGTCCACTTCAGCTTCTACATCG |
|  |  | *GmCOL5*-1R | CAAGAACTTGTGCACGCCACTAAC |
| *GmCOL6* | *GmCOL6* | *GmCOL6*-1F | AGAAATGGATCGACGGGACC |
|  |  | *GmCOL6*-1R | GTTATGGAAACCCAACTGCCA |
| *GmCOL8* | *GmCOL8* | *GmCOL8*-1F | TGCTGGTCTGAGCGAACCATTTAC |
|  |  | *GmCOL8*-1R | TGTTGGCACTAGTGGTGTTCCTCT |
| *GmCOL9* | *GmCOL9* | *GmCOL9*-1F | GACACATACCCTGCTATCCAATCC |
|  |  | *GmCOL9*-1R | CTCCTGACCCATCAATGGGAACTT |
|  |  | *GmCOL9*-2F | CTGCAGCTTTGGGAGTAGTTGATAGG |
|  |  | *GmCOL9*-2R | AGTACATCTTCCCACGTCACTTAAAAGTC |
| *GmCOL10* | *GmCOL10* | *GmCOL10*-1F | CCGCACCCAAAGAAATCAACTCAC |
|  |  | *GmCOL10*-1R | GGAACCATATTCAAGTCATGTCCTCAACTC |
| *GmCOL13* | *GmCOL13* | *GmCOL13*-1F | TCGACGGATGTCATTGTACCTAGATGTG |
|  |  | *GmCOL13*-1R | GCAATACGAACAAAGCCTCAGCATC |
| *GmCOL14* | *GmCOL14* | *GmCOL14*-1F | CAGGGCTTAGCCGCTTGTT |
|  |  | *GmCOL14*-1R | CACTGGAGTGGCCAGATTAAAA |
| *GmCOL15* | *GmCOL15* | *GmCOL15*-1F | GTGTCCTTTGCTTGTGCACTTACC |
|  |  | *GmCOL15*-1R | GCAAAACCAATCACCGTCACCATC |
|  |  | *GmCOL15*-2F | TCAGGCATCAGCACCAACTTTGAC |
|  |  | *GmCOL15*-2R | CGTGAACCAGAGGCTGATATCTTTCC |

**Table S1** Continued

| **Gene** | **Template** | **Primer Name** | **Sequence (5’3’)** |
| --- | --- | --- | --- |
| *GmCOL16* | *GmCOL16* | *GmCOL16*-1F | CGCACTCACCCACCTTTCTTATCA |
|  |  | *GmCOL16*-1R | CGTGCCAGGACGTTATTGGTTGAA |
|  |  | *GmCOL16*-2F | GGATGTCAATGGAGTATGCGAATGC |
|  |  | *GmCOL16*-2R | GTTGGTGCCATTGTACGGTATGGA |
| *GmCOL19* | *GmCOL19* | *GmCOL19*-1F | GAAAGCAAGACAGACCCCACAGTA |
|  |  | *GmCOL19*-1R | GCTACCCCTTTCCATAACTACGCTTCCA |
| *GmCOL20* | *GmCOL20* | *GmCOL20*-1F | GGTGTTGCTGTCCTTGAACAATGG |
|  |  | *GmCOL20*-1R | TGGGAATTGCAGAGGAAGGGATTG |
|  |  | *GmCOL20*-2F | TCGCGATAACCCGCAGTCTTGATA |
|  |  | *GmCOL20*-2R | GTGGTTGGGAATCGGTTGGTACTT |
| *GmCOL22* | *GmCOL22* | *GmCOL22*-1F | GTGAGCCAAGATGAGTTGAACCGA |
|  |  | *GmCOL22*-1R | ATGCACTCCTAACCCAACTGTGAC |
| *GmCOL23* | *GmCOL23* | *GmCOL23*-1F | ACCGACAAACTTGGGAGAACCAAC |
|  |  | *GmCOL23*-1R | TCAAAGCTCCAGCCAATGAGCTTTGCG |
| *GmCOL24* | *GmCOL24* | *GmCOL24*-1F | CCACAGACATGCTACAGGTTCCAA |
|  |  | *GmCOL24*-1R | TGATCGATGAAGAGGCCTTGACTG |
| *GmCOL25* | *GmCOL25* | *GmCOL25*-1F | ACAGTAGGGACTCAGGGGTGATTG |
|  |  | *GmCOL25*-1R | TGGATTAGGCGTAGCTCGTGCATA |
| *GmCOL26* | *GmCOL26* | *GmCOL26*-1F | CCTCCACCTCAAAGTTCCATAACCAC |
|  |  | *GmCOL26*-1R | CACTGCCATTCTAGAGCCTCAACA |
| *GmCOL28* | *GmCOL28* | *GmCOL28*-1F | GTGTCACTCCAAACACTGCAGGAT |
|  |  | *GmCOL28*-1R | CCTTCCGATACGTCACCATTGAGA |
|  |  | *GmCOL28*-2F | CGTATGGAGTAGCTTTAGGAGTAGGAG |
|  |  | *GmCOL28*-2R | GGATCGGTGACTTGCCTTTTAC |
