# Supplementary material for: CONSTANS Polymorphism Modulates Flowering Time and Maturity in Soybean
Source: Front Plant Sci. 2022 Mar 17;13:817544. doi: 10.3389/fpls.2022.817544 (PMC8969907; doi:10.3389/fpls.2022.817544)
Supplement: Supplementary file 4 [file Table_2.docx]

**Table S2** Polymorphic sites used for the haplotypes of 20 soybean *COL* family genes

| **Gene** | **Polymorphic sites of haplotypes** |
| --- | --- |
| ***GmCOL2*** | s378, _Indel_382, s385, s386, s387, _Indel_389, s400, s485, **s744**, _Indel_918, _Indel_919, _Indel_920, **s958**, **s1138**, **s1139**, **s1218**, **s1260**, _Indel_1336, _Indel_1337, _Indel_1419, s1425, s1428, s1433, _Indel_1447, s1477, s1492, s1501, s1513, s1560, s1611, s1625, s1687, s1746, s1776, s1781, s1796, _Indel_1822, s1877, s1896, s2147, s2301, s2440, _Indel_2473, _Indel_2567, **s3207**, s3302 |
| ***GmCOL4*** | **s644**, _Indel_787, _Indel_788, s822, s877, s1113, s1222, s1282, s1290, **s1566**, **s1954**, s2232, s2234, _Indel_2235, _Indel_2236, _Indel_2237, _Indel_2238, _Indel_2239, _Indel_2240, _Indel_2241, _Indel_2242, _Indel_2243, _Indel_2244, _Indel_2245, _Indel_2246, _Indel_2247, _Indel_2248, _Indel_2249, _Indel_2250, _Indel_2251, _Indel_2252, _Indel_2253, _Indel_2254, _Indel_2255, _Indel_2256, _Indel_2257, _Indel_2258, s2259, s2260, s2534, s2584, _Indel_2774, s2784, _Indel_2785, _Indel_2786, s2787, s2790, s2829, s2918, s2973, s3066, s3088, s3091, s3093, s3300, s3355, s3409, s3442, s3484, s3536, s3580, s3764, s3921, s3946, s4175, s4510, s4567, s4586, s4599, s4697, s4795, s5105, s5176, s5495, s5501, s5513, s5524, s5536, s5549, s5569, s5586, s5587, s5800, s5970, s6004, s6198, s6552, s6603, s6648, s6674, s6808, s6822, s7084, s7103, s7313, s7345, s7607, _Indel_7633, _Indel_7634, _Indel_7635, _Indel_7636, _Indel_7637, s7668, s7723, s7745, s7754, s7790, s7809, _Indel_8663, s9073, **s10932**, **s10984**, _Indel_11280, s11283, s11415, s11418, s11501, s11502, _Indel_11503, _Indel_11504, s11524 |
| ***GmCOL5*** | _Indel_394, _Indel_395, _Indel_396-410, s428, **s986**, **s999**, **s1052**, _Indel_1256, _Indel_1257, _Indel_1280, s1302, s1304, _Indel_1305, s1310, s1393, s1480, _Indel_1549, _Indel_1550, s1562, s1576, s1625, _Indel_1633-1651, _Indel_1652, _Indel_1653, _Indel_1654, _Indel_1655-1715, _s-Indel_1716, _Indel_1717-1767, s1768, _Indel_1769, _Indel_1770, _Indel_1771, _Indel_1772, _Indel_1773, _Indel_1774-1792, _s-Indel_1793, _Indel_1794-1837, _s-Indel_1838, _s-Indel_1839-2025, s2041, s2108, s2185, s2191, **s2352** |
| ***GmCOL6*** | s304, s348, _Indel_498, s499, s508, _Indel_511, **s924**, **s1005**, _Indel_1058, **s1065**, **s1083**, _Indel_1116, s1117, _Indel_1280, _Indel_1281, _Indel_1282, _Indel_1319-1378, **s1481**, **s1707**, s1791, s1834, _Indel_1968, _Indel_1969, _Indel_1070, s1979, s1996, _Indel_2045, _Indel_2046, s2083, s2345, **s2570**, s2571, _Indel_2377-2395, _Indel_2745, s2977, s2988, s3001 |
| ***GmCOL8*** | s458, **s714**, **s999**, s2698, s2856 |
| ***GmCOL9*** | **s791**, **s824**, **s905**, _Indel_1290, s1457, s1529, _Indel_1552, _Indel_1597, _Indel_1598, _Indel_1599, _Indel_1600, _Indel_1601, _Indel_1602, _Indel_1603, _Indel_2099, s2361, s2365, s2601, s2738, s3508, **s4186**, s4239, _Indel_4240, **s4654**, **s4960**, _Indel_5107, s5270, s5405, s5493, s5549 |
| ***GmCOL10*** | **s575**, **s1664**, **s1827**, _Indel_2215, _Indel_2216, _Indel_2217, _Indel_2218, s2413, _Indel_2566, _Indel_2567 |
| ***GmCOL13*** | **s584**, **s1946**, **s1989**, **s2049** |
| ***GmCOL14*** | _Indel_198, _Indel_645, _Indel_910, _Indel_1047, **s1134**, _Indel_1178, _Indel_1205, **s1538**, _Indel_1642, _Indel_1662, _Indel_1918, **s1921**, _Indel_2095, _Indel_2446, _Indel_2447, _Indel_2462, _Indel_2479, _Indel_2523, _Indel_2524 |
| ***GmCOL15*** | **s2910**, **s3278**, **s3867** |
| ***GmCOL16*** | s124, _Indel_130, **s525**, **s547**, **s590**, **s935**, **s1161**, s1574, s1623, s1700, _Indel_1810, _Indel_1811, s1888, s1948, s2167, _Indel_2184, _Indel_2196, _Indel_2197, s2221, s2250, s2405, s2651, s2773, s2785, s2825, s2909, s2931, _Indel_3297, _Indel_3298, _Indel_3299, _Indel_3300, _Indel_3301, _Indel_3302, _Indel_3303, _Indel_3304, s3920, **s4250**, **s4366**, **s4490**, _Indel_4572, _Indel_4573, s4719, s4752, s4773, s4854 |
| ***GmCOL19*** | **s491**, **s515**, **s910**, **s933**, s1719, s2568, s2570 |
| ***GmCOL20*** | s1310, **s1484**, s1605, s1662, s2093, _Indel_2448, _Indel_2449, _Indel_2450, s2455, s2492, s2526, **s2653**, **s2655**, **s2692**, s3066, s3149, s3197, s3201, s3247, s3361, s4250, s4303, s4362, s4363, s4401, **s4507**, _Indel_4897, _Indel_4898, _Indel_4899, _Indel_4900, _Indel_4901, _Indel_4902, _Indel_4903, _Indel_4904, _Indel_4905, _Indel_4906, _Indel_4907, _Indel_4908, _Indel_4909, **s4997**, **s5051** |
| ***GmCOL22*** | _Indel_534, **s760**, _Indel_841-848, _Indel_849-851, _Indel_852, s995, s1082, **s1183**, **s1198**, _Indel_1227, _Indel_1249, **s1268**, **s1417**, **s1434**, _Indel_1670-1675, s1681, _Indel_1690-1694, s1734, s1756, s1893, s2062, **s2201**, **s2441**, **s2451**, **s2495**, s2772, s2774, s2775, s2786, s2796, s2899, s2973, s2975 |
| ***GmCOL23*** | s429, _Indel_536, **s681**, s1019, **s1037**, _Indel_1071, _Indel_1072, _Indel_1073, _Indel_1074, _Indel_1075, _Indel_1076, **s1086**, **s1196**, **s1226**, _Indel_1858, **s1911**, s2116, s2137, s2146, _Indel_2153, _Indel_2159, s2169, _Indel_2264, s2277, _Indel_2301, _s-Indel_2302, s2395, _Indel_2472, s2511, **s2520**, _Indel_2823, s3017 |

**Note:** Red color means critical polymorphic sites of tagging haplotype**.**

**Table S2** Continued

| **Gene** | **Polymorphic sites of haplotypes** |
| --- | --- |
| ***GmCOL24*** | s250, s278, s374, _Indel_444, _Indel_480, _Indel_481, _Indel_482, s533, **s682**, **s706**, **s763**, **s813**, **s816**, **s872**, **s1058**, s1216, s1295, s1336, s1410, s1437, s1868, s1943, s2095, _Indel_2156 |
| ***GmCOL25*** | s659, s857, s928, **s1315**, **s1338**, _Indel_1344, _Indel_1345, _Indel_1346, _Indel_1347, _Indel_1348, _Indel_1349, _Indel_1350, _Indel_1351, _Indel_1352, _Indel_1353, **s1380**, **s1453**, **s1562**, **s1600**, **s1603**, **s1614**, **s1616**, **s1643**, s1702, s1704, s1775, s1870, s1909, s1913, s1960, s1984, _Indel_2030 |
| ***GmCOL26*** | s475, **s523**, **s573**, **s575**, **s615**, **s624**, **s625**, **s650**, **s663**, **s675**, **s679**, **s748**, **s783**, **s860**, **s881**, **s884**, **s885**, s1092, s1208, s1209, s1262, s1303, s1349, s1359 |
| ***GmCOL28*** | _Indel_330, s794, **s813, s1066**, _Indel_1281, _Indel_1282, _Indel_1283, _Indel_1284, s1725, s1812, s2055, s2784, s2786, _Indel_2788, _Indel_2789, _s-Indel_2790, s2881, _Indel_2928, s3122, s3294, s3380, s3416, _Indel_3451, s3477, s3759, s3773, _Indel_3811, s3883, _Indel_3983, _Indel_4003, s4016, _Indel_4043, _Indel_4199, s4207, s4292, s4537, s4575, _Indel_4598 |

**Note:** Red font means critical polymorphic sites of tagging haplotype**.**
