# Supplementary material for: CONSTANS Polymorphism Modulates Flowering Time and Maturity in Soybean
Source: Front Plant Sci. 2022 Mar 17;13:817544. doi: 10.3389/fpls.2022.817544 (PMC8969907; doi:10.3389/fpls.2022.817544)
Supplement: Supplementary file 5 [file Table_3.docx]

Table S3 Haplotypes of 20 soybean *GmCOL* family genes in 128 varieties covering 14 maturity groups

| **Variety Name** | *GmCOL2* | *GmCOL4* | *GmCOL5* | *GmCOL6* | *GmCOL8* | *GmCOL9* | *GmCOL10* | *GmCOL13* | *GmCOL14* | *GmCOL15* | *GmCOL16* | *GmCOL19* | *GmCOL20* | *GmCOL22* | *GmCOL23* | *GmCOL24* | *GmCOL25* | *GmCOL26* | *GmCOL28* |
| --- | --- | --- | --- | --- | --- | --- | --- | --- | --- | --- | --- | --- | --- | --- | --- | --- | --- | --- | --- |
| Star4/75^0000^ | *Hap3* | *Hap1* | ND | *Hap1* | ND | *Hap2* | *Hap1* | *Hap1* | ND | ND | ND | ND | *Hap2* | *Hap1* | *Hap8* | *Hap1* | *Hap1* | *Hap3* | ND |
| Hujiao07-2479^0000^ | *Hap2* | *Hap1* | *Hap1* | *Hap2* | *Hap1* | *Hap3* | *Hap2* | *Hap1* | *Hap1* | *Hap1* | *Hap3* | *Hap1* | *Hap2* | *Hap1* | *Hap1* | ND | *Hap1* | *Hap1* | ND |
| Hujiao07-2123^0000^ | *Hap2* | *Hap1* | ND | *Hap2* | ND | *Hap8* | ND | *Hap1* | *Hap1* | ND | *Hap3* | ND | *Hap3* | *Hap1* | ND | *Hap1* | ND | *Hap1* | *Hap1* |
| Dongnong36^0000^ | *Hap5* | ND | *Hap1* | *Hap1* | *Hap1* | *Hap1* | *Hap1* | *Hap1* | *Hap1* | *Hap1* | *Hap3* | *Hap1* | ND | *Hap1* | *Hap1* | *Hap3* | *Hap1* | *Hap2* | ND |
| Paula^0000^ | *Hap2* | *Hap1* | *Hap1* | *Hap1* | *Hap1* | *Hap1* | *Hap1* | *Hap1* | *Hap1* | *Hap1* | *Hap1* | *Hap1* | *Hap2* | *Hap1* | *Hap2* | *Hap2* | *Hap1* | *Hap2* | *Hap2* |
| R-4^0000^ | *Hap4* | *Hap1* | *Hap2* | *Hap1* | *Hap1* | *Hap1* | *Hap2* | *Hap1* | ND | *Hap1* | *Hap4* | *Hap1* | *Hap2* | *Hap5* | *Hap1* | *Hap1* | *Hap1* | *Hap2* | *Hap2* |
| Dongnong41^0000^ | *Hap2* | *Hap1* | *Hap1* | *Hap4* | ND | ND | ND | *Hap1* | *Hap1* | ND | ND | *Hap1* | *Hap3* | *Hap3* | ND | *Hap5* | ND | *Hap3* | *Hap2* |
| Lingbei8^0000^ | *Hap2* | *Hap1* | *Hap2* | *Hap2* | *Hap1* | ND | *Hap1* | *Hap1* | *Hap1* | *Hap1* | *Hap2* | *Hap1* | *Hap2* | *Hap1* | *Hap1* | *Hap1* | *Hap1* | *Hap2* | *Hap3* |
| Dongnong 41-C^0000^ | *Hap2* | *Hap1* | ND | *Hap2* | ND | ND | ND | *Hap1* | *Hap1* | *Hap1* | *Hap2* | ND | *Hap2* | *Hap1* | *Hap1* | *Hap1* | *Hap1* | *Hap2* | *Hap3* |
| Maple-Presto^000^ | *Hap2* | *Hap1* | *Hap1* | *Hap1* | *Hap1* | *Hap3* | *Hap1* | *Hap1* | ND | *Hap1* | *Hap3* | *Hap1* | *Hap6* | *Hap1* | *Hap1* | ND | *Hap1* | *Hap1* | *Hap3* |
| OAC-Vision^000^ | *Hap1* | *Hap1* | *Hap1* | *Hap1* | ND | *Hap1* | *Hap1* | *Hap1* | *Hap1* | *Hap1* | *Hap1* | *Hap1* | *Hap2* | *Hap1* | *Hap1* | *Hap1* | *Hap1* | *Hap2* | ND |
| Rassvet^000^ | *Hap4* | *Hap1* | *Hap1* | *Hap1* | *Hap1* | *Hap2* | *Hap1* | *Hap1* | *Hap1* | *Hap1* | *Hap5* | *Hap1* | *Hap3* | *Hap1* | *Hap1* | *Hap1* | *Hap1* | *Hap2* | ND |
| Jug-30^000^ | *Hap5* | *Hap1* | *Hap2* | *Hap1* | *Hap1* | *Hap1* | *Hap1* | *Hap1* | *Hap1* | *Hap1* | *Hap7* | *Hap1* | *Hap2* | *Hap1* | *Hap3* | ND | *Hap1* | *Hap2* | *Hap2* |
| Mageva^000^ | *Hap2* | *Hap1* | *Hap2* | *Hap1* | *Hap1* | *Hap2* | *Hap2* | *Hap1* | *Hap1* | *Hap1* | *Hap1* | *Hap1* | *Hap2* | *Hap3* | *Hap4* | *Hap2* | *Hap1* | *Hap2* | ND |
| R2^000^ | *Hap5* | *Hap1* | *Hap2* | *Hap1* | ND | *Hap2* | *Hap2* | *Hap1* | *Hap1* | *Hap1* | *Hap4* | *Hap1* | *Hap3* | *Hap1* | *Hap2* | ND | *Hap1* | *Hap2* | *Hap2* |
| Heihe35^000^ | *Hap4* | *Hap1* | *Hap2* | *Hap2* | *Hap1* | *Hap2* | *Hap1* | *Hap1* | *Hap1* | *Hap2* | *Hap2* | *Hap1* | *Hap2* | *Hap1* | *Hap1* | *Hap1* | *Hap1* | *Hap2* | *Hap3* |

**ND: Not available dataTable** **S3** Continued

| Variety Name | *GmCOL2* | *GmCOL4* | *GmCOL5* | *GmCOL6* | *GmCOL8* | *GmCOL9* | *GmCOL10* | *GmCOL13* | *GmCOL14* | *GmCOL15* | *GmCOL16* | *GmCOL19* | *GmCOL20* | *GmCOL22* | *GmCOL23* | *GmCOL24* | *GmCOL25* | *GmCOL26* | *GmCOL28* |
| --- | --- | --- | --- | --- | --- | --- | --- | --- | --- | --- | --- | --- | --- | --- | --- | --- | --- | --- | --- |
| Canatto^00^ | *Hap4* | *Hap1* | *Hap2* | *Hap1* | *Hap1* | *Hap9* | *Hap1* | *Hap1* | *Hap1* | *Hap1* | *Hap1* | *Hap1* | *Hap2* | *Hap1* | *Hap1* | *Hap1* | *Hap1* | *Hap1* | *Hap2* |
| Maple-Ridge^00^ | *Hap1* | *Hap1* | *Hap1* | *Hap3* | *Hap1* | *Hap3* | *Hap1* | *Hap1* | *Hap1* | *Hap1* | *Hap1* | *Hap1* | *Hap3* | *Hap1* | *Hap2* | *Hap1* | *Hap1* | *Hap2* | ND |
| Daksoy^00^ | *Hap1* | *Hap1* | *Hap1* | *Hap1* | *Hap1* | *Hap1* | *Hap1* | *Hap1* | *Hap1* | *Hap1* | *Hap3* | *Hap1* | *Hap3* | *Hap1* | *Hap1* | *Hap1* | *Hap1* | *Hap1* | ND |
| McCall^00^ | *Hap1* | *Hap1* | *Hap1* | *Hap3* | *Hap1* | *Hap3* | *Hap1* | *Hap1* | *Hap1* | *Hap1* | *Hap1* | *Hap1* | *Hap3* | *Hap1* | *Hap1* | *Hap1* | *Hap1* | *Hap1* | ND |
| Agassiz^00^ | *Hap1* | *Hap1* | *Hap1* | *Hap1* | ND | ND | ND | *Hap1* | *Hap1* | *Hap1* | *Hap1* | *Hap1* | *Hap4* | *Hap1* | ND | *Hap1* | ND | *Hap1* | *Hap3* |
| Mengdou32^00^ | *Hap3* | *Hap1* | *Hap1* | *Hap2* | *Hap1* | *Hap4* | *Hap1* | *Hap1* | *Hap1* | *Hap1* | *Hap1* | *Hap2* | *Hap2* | *Hap1* | *Hap3* | ND | *Hap1* | *Hap2* | *Hap3* |
| Beidou16^00^ | *Hap3* | ND | ND | *Hap2* | ND | *Hap10* | ND | *Hap1* | *Hap1* | *Hap1* | *Hap1* | *Hap1* | ND | *Hap1* | ND | ND | *Hap1* | *Hap1* | *Hap1* |
| Dongnong44^00^ | *Hap6* | *Hap1* | *Hap1* | *Hap2* | *Hap1* | *Hap7* | *Hap1* | *Hap1* | *Hap1* | *Hap1* | *Hap1* | *Hap1* | *Hap3* | *Hap1* | *Hap1* | *Hap1* | *Hap1* | *Hap2* | *Hap3* |
| Mengdou11^00^ | *Hap2* | *Hap1* | *Hap1* | *Hap2* | *Hap1* | *Hap4* | *Hap2* | *Hap1* | *Hap1* | *Hap1* | *Hap1* | *Hap2* | *Hap2* | *Hap1* | *Hap1* | *Hap1* | *Hap1* | *Hap1* | *Hap1* |
| Traill^0^ | *Hap1* | *Hap1* | *Hap1* | *Hap1* | ND | *Hap1* | *Hap1* | *Hap1* | *Hap1* | *Hap1* | *Hap1* | *Hap1* | *Hap2* | *Hap3* | *Hap10* | *Hap1* | ND | *Hap2* | ND |
| Chico^0^ | *Hap2* | *Hap1* | *Hap1* | *Hap1* | *Hap1* | ND | *Hap2* | *Hap1* | *Hap1* | ND | ND | *Hap1* | *Hap3* | *Hap1* | ND | *Hap1* | *Hap1* | *Hap2* | ND |
| Barnes^0^ | *Hap2* | *Hap1* | *Hap1* | *Hap1* | *Hap1* | *Hap1* | *Hap3* | *Hap1* | *Hap1* | *Hap1* | *Hap1* | *Hap1* | *Hap5* | *Hap1* | *Hap1* | *Hap1* | *Hap1* | *Hap2* | *Hap2* |
| Norpro^0^ | *Hap1* | *Hap1* | *Hap1* | *Hap3* | *Hap1* | *Hap1* | *Hap2* | *Hap1* | ND | *Hap1* | *Hap1* | *Hap1* | *Hap2* | *Hap1* | *Hap1* | *Hap1* | *Hap5* | *Hap1* | *Hap1* |
| Dawson^0^ | *Hap2* | *Hap1* | ND | *Hap3* | *Hap1* | *Hap1* | *Hap1* | *Hap1* | *Hap1* | *Hap1* | *Hap1* | *Hap1* | *Hap2* | *Hap1* | *Hap1* | *Hap1* | *Hap1* | *Hap1* | *Hap3* |
| Jiangmodou 1^0^ | *Hap2* | ND | *Hap1* | *Hap2* | *Hap1* | ND | *Hap1* | *Hap1* | *Hap1* | *Hap1* | *Hap1* | *Hap1* | ND | *Hap1* | *Hap3* | ND | *Hap1* | *Hap3* | *Hap1* |
| Heihe18^0^ | *Hap4* | *Hap1* | *Hap2* | *Hap2* | *Hap1* | *Hap4* | *Hap2* | *Hap1* | *Hap1* | *Hap2* | *Hap2* | *Hap1* | *Hap4* | *Hap1* | *Hap1* | *Hap1* | *Hap1* | *Hap2* | *Hap1* |

**ND: Not available data**

**Table** **S3** Continued

| Variety Name | *GmCOL2* | *GmCOL4* | *GmCOL5* | *GmCOL6* | *GmCOL8* | *GmCOL9* | *GmCOL10* | *GmCOL13* | *GmCOL14* | *GmCOL15* | *GmCOL16* | *GmCOL19* | *GmCOL20* | *GmCOL22* | *GmCOL23* | *GmCOL24* | *GmCOL25* | *GmCOL26* | *GmCOL28* |
| --- | --- | --- | --- | --- | --- | --- | --- | --- | --- | --- | --- | --- | --- | --- | --- | --- | --- | --- | --- |
| Heihe43^0^ | *Hap4* | *Hap1* | ND | *Hap2* | ND | ND | *Hap2* | *Hap1* | ND | *Hap2* | *Hap3* | *Hap1* | *Hap5* | *Hap1* | *Hap5* | *Hap1* | *Hap1* | *Hap2* | *Hap3* |
| Heihe27^0^ | *Hap4* | *Hap1* | *Hap1* | *Hap2* | *Hap1* | *Hap4* | *Hap1* | *Hap1* | *Hap1* | *Hap2* | *Hap1* | *Hap1* | *Hap4* | *Hap5* | *Hap6* | *Hap1* | *Hap1* | *Hap1* | *Hap1* |
| Beidou37^0^ | *Hap2* | *Hap1* | *Hap1* | *Hap2* | *Hap1* | *Hap5* | *Hap1* | *Hap1* | *Hap1* | *Hap1* | *Hap1* | *Hap1* | *Hap2* | *Hap1* | ND | *Hap1* | *Hap1* | *Hap2* | ND |
| Dengke1^0^ | *Hap2* | *Hap1* | *Hap1* | *Hap2* | *Hap1* | *Hap11* | *Hap1* | *Hap1* | *Hap1* | *Hap1* | *Hap1* | *Hap1* | *Hap3* | *Hap1* | *Hap9* | *Hap1* | *Hap1* | *Hap3* | *Hap1* |
| Fengshou12^0^ | *Hap2* | *Hap1* | ND | ND | ND | ND | ND | *Hap1* | *Hap1* | *Hap1* | *Hap4* | ND | *Hap2* | ND | ND | ND | ND | ND | *Hap3* |
| Dongnong-4^0^ | *Hap2* | *Hap1* | *Hap2* | *Hap1* | *Hap1* | *Hap1* | *Hap1* | *Hap1* | *Hap1* | *Hap1* | *Hap1* | *Hap1* | ND | *Hap1* | *Hap7* | *Hap1* | *Hap1* | *Hap2* | *Hap3* |
| Hefeng25^0^ | *Hap2* | ND | *Hap1* | *Hap2* | *Hap1* | *Hap5* | *Hap1* | *Hap1* | *Hap1* | *Hap2* | *Hap1* | *Hap1* | ND | *Hap3* | ND | *Hap1* | *Hap1* | *Hap2* | *Hap1* |
| Haroson ^I^ | *Hap1* | *Hap1* | *Hap1* | *Hap3* | *Hap1* | *Hap1* | *Hap1* | *Hap1* | *Hap1* | *Hap1* | *Hap1* | *Hap1* | *Hap3* | *Hap1* | *Hap1* | *Hap1* | *Hap1* | *Hap1* | *Hap3* |
| Kato ^I^ | *Hap1* | *Hap1* | *Hap2* | *Hap1* | *Hap1* | *Hap1* | *Hap1* | *Hap1* | *Hap1* | *Hap1* | *Hap1* | ND | *Hap1* | *Hap1* | *Hap1* | *Hap1* | ND | *Hap1* | *Hap1* |
| Parker ^I^ | *Hap2* | *Hap1* | *Hap1* | *Hap5* | *Hap1* | *Hap1* | *Hap1* | *Hap1* | *Hap1* | *Hap1* | *Hap1* | *Hap1* | *Hap2* | *Hap1* | *Hap1* | *Hap1* | *Hap2* | *Hap1* | *Hap1* |
| Granite ^I^ | *Hap2* | *Hap1* | *Hap2* | *Hap1* | *Hap1* | *Hap1* | *Hap1* | *Hap1* | *Hap1* | *Hap1* | *Hap1* | *Hap1* | *Hap1* | *Hap1* | *Hap1* | *Hap1* | *Hap1* | *Hap1* | *Hap1* |
| NE1900 ^I^ | *Hap2* | ND | *Hap1* | *Hap1* | *Hap1* | *Hap1* | *Hap1* | *Hap1* | *Hap1* | *Hap1* | *Hap2* | *Hap1* | *Hap1* | *Hap1* | *Hap1* | *Hap1* | ND | *Hap2* | *Hap3* |
| Heinong16 ^I^ | *Hap3* | *Hap1* | *Hap2* | *Hap1* | ND | *Hap1* | ND | *Hap1* | *Hap1* | *Hap1* | *Hap1* | *Hap1* | *Hap3* | *Hap1* | ND | *Hap1* | *Hap1* | *Hap3* | *Hap3* |
| Taixingheidou ^I^ | *Hap3* | *Hap1* | ND | ND | ND | *Hap2* | *Hap1* | *Hap1* | *Hap1* | *Hap1* | *Hap1* | *Hap1* | *Hap2* | *Hap1* | *Hap1* | *Hap1* | ND | *Hap2* | *Hap1* |
| Heinong26 ^I^ | *Hap3* | *Hap1* | *Hap2* | *Hap2* | *Hap1* | *Hap1* | *Hap1* | *Hap1* | *Hap1* | *Hap1* | *Hap1* | *Hap1* | *Hap2* | *Hap1* | *Hap1* | ND | *Hap1* | *Hap2* | *Hap1* |
| Suinong14 ^I^ | *Hap6* | *Hap1* | *Hap1* | *Hap2* | *Hap1* | *Hap2* | *Hap1* | *Hap1* | *Hap1* | *Hap1* | *Hap1* | *Hap1* | *Hap2* | *Hap1* | *Hap1* | ND | *Hap1* | *Hap2* | *Hap1* |

**ND: Not available data**

**Table** **S3** Continued

| Variety Name | *GmCOL2* | *GmCOL4* | *GmCOL5* | *GmCOL6* | *GmCOL8* | *GmCOL9* | *GmCOL10* | *GmCOL13* | *GmCOL14* | *GmCOL15* | *GmCOL16* | *GmCOL19* | *GmCOL20* | *GmCOL22* | *GmCOL23* | *GmCOL24* | *GmCOL25* | *GmCOL26* | *GmCOL28* |
| --- | --- | --- | --- | --- | --- | --- | --- | --- | --- | --- | --- | --- | --- | --- | --- | --- | --- | --- | --- |
| Holt ^II^ | *Hap4* | *Hap1* | *Hap1* | *Hap1* | *Hap1* | *Hap1* | *Hap1* | *Hap1* | *Hap1* | *Hap1* | *Hap1* | *Hap1* | *Hap3* | *Hap1* | *Hap1* | *Hap1* | *Hap1* | *Hap2* | *Hap1* |
| Olympus ^II^ | *Hap10* | *Hap1* | *Hap1* | *Hap1* | ND | *Hap1* | *Hap1* | *Hap1* | *Hap1* | *Hap1* | *Hap3* | *Hap1* | *Hap1* | *Hap1* | *Hap1* | *Hap1* | ND | *Hap1* | *Hap1* |
| Century-84 ^II^ | *Hap1* | *Hap1* | *Hap2* | *Hap1* | *Hap1* | *Hap1* | *Hap1* | *Hap1* | *Hap1* | *Hap1* | *Hap8* | *Hap1* | *Hap1* | *Hap1* | *Hap1* | ND | *Hap1* | *Hap1* | *Hap1* |
| IL1 ^II^ | *Hap2* | *Hap1* | *Hap2* | *Hap1* | *Hap1* | *Hap2* | *Hap1* | *Hap1* | *Hap1* | *Hap1* | *Hap8* | *Hap1* | *Hap2* | *Hap1* | *Hap1* | ND | *Hap3* | *Hap2* | *Hap1* |
| LN92-7369 ^II^ | ND | *Hap1* | *Hap2* | *Hap1* | *Hap1* | *Hap1* | *Hap1* | *Hap1* | *Hap1* | *Hap1* | *Hap3* | *Hap1* | *Hap1* | *Hap1* | *Hap1* | ND | *Hap1* | *Hap1* | *Hap1* |
| Jilin20 ^II^ | *Hap4* | *Hap1* | *Hap2* | *Hap2* | *Hap1* | ND | *Hap2* | *Hap1* | *Hap1* | *Hap1* | *Hap4* | *Hap2* | *Hap2* | *Hap1* | *Hap1* | *Hap1* | *Hap1* | *Hap2* | *Hap1* |
| Yongchengzihuadou ^II^ | *Hap3* | *Hap2* | *Hap3* | *Hap2* | *Hap1* | *Hap1* | *Hap1* | *Hap1* | *Hap1* | *Hap1* | *Hap2* | *Hap1* | *Hap9* | *Hap2* | ND | ND | *Hap1* | *Hap2* | *Hap2* |
| Xiangchundou24 ^II^ | *Hap8* | *Hap4* | *Hap2* | *Hap2* | *Hap2* | ND | *Hap1* | *Hap1* | ND | *Hap1* | *Hap2* | ND | *Hap2* | *Hap2* | ND | *Hap2* | *Hap1* | *Hap3* | *Hap1* |
| Tiefeng19 ^II^ | ND | *Hap1* | ND | *Hap2* | *Hap1* | *Hap1* | ND | *Hap2* | *Hap1* | *Hap1* | *Hap2* | *Hap2* | *Hap1* | *Hap1* | ND | *Hap1* | *Hap4* | *Hap2* | *Hap1* |
| Athow ^III^ | *Hap1* | *Hap1* | *Hap1* | *Hap1* | ND | *Hap1* | ND | *Hap1* | ND | *Hap1* | *Hap3* | *Hap2* | *Hap1* | *Hap1* | ND | *Hap1* | *Hap1* | *Hap1* | *Hap3* |
| Zhonghuang13 ^III^ | *Hap2* | *Hap3* | *Hap2* | *Hap2* | *Hap2* | *Hap1* | *Hap1* | *Hap1* | *Hap1* | *Hap1* | ND | *Hap1* | *Hap2* | *Hap1* | *Hap1* | *Hap1* | *Hap1* | *Hap2* | *Hap1* |
| Zhonghuang30 ^III^ | *Hap2* | *Hap1* | *Hap2* | *Hap1* | *Hap1* | ND | *Hap1* | *Hap1* | *Hap1* | *Hap1* | *Hap1* | *Hap3* | *Hap1* | *Hap1* | *Hap1* | *Hap1* | *Hap1* | *Hap1* | *Hap1* |
| LN89-5699 ^III^ | *Hap4* | *Hap1* | *Hap1* | *Hap1* | *Hap1* | *Hap1* | *Hap1* | *Hap1* | *Hap1* | *Hap1* | *Hap1* | *Hap4* | *Hap8* | *Hap1* | *Hap1* | *Hap1* | *Hap1* | *Hap1* | *Hap1* |
| KS3494 ^III^ | *Hap2* | *Hap1* | *Hap1* | *Hap1* | *Hap1* | *Hap1* | *Hap1* | *Hap1* | *Hap1* | *Hap1* | *Hap1* | *Hap1* | *Hap2* | *Hap1* | *Hap1* | *Hap1* | *Hap2* | *Hap1* | *Hap1* |
| IL2 ^III^ | *Hap3* | *Hap2* | ND | *Hap1* | *Hap1* | *Hap1* | *Hap1* | *Hap1* | *Hap1* | *Hap1* | *Hap3* | *Hap1* | *Hap3* | *Hap1* | *Hap1* | *Hap4* | *Hap1* | *Hap2* | *Hap1* |
| Williams-82 ^III^ | *Hap1* | *Hap1* | *Hap1* | *Hap1* | *Hap1* | *Hap1* | *Hap1* | *Hap1* | *Hap1* | *Hap1* | *Hap1* | *Hap1* | *Hap1* | *Hap1* | *Hap1* | *Hap1* | *Hap1* | *Hap1* | *Hap1* |

**ND: Not available dataTable** **S3** Continued

| Variety Name | *GmCOL2* | *GmCOL4* | *GmCOL5* | *GmCOL6* | *GmCOL8* | *GmCOL9* | *GmCOL10* | *GmCOL13* | *GmCOL14* | *GmCOL15* | *GmCOL16* | *GmCOL19* | *GmCOL20* | *GmCOL22* | *GmCOL23* | *GmCOL24* | *GmCOL25* | *GmCOL26* | *GmCOL28* |
| --- | --- | --- | --- | --- | --- | --- | --- | --- | --- | --- | --- | --- | --- | --- | --- | --- | --- | --- | --- |
| Tiefeng33 ^III^ | *Hap4* | *Hap1* | *Hap2* | *Hap1* | *Hap1* | *Hap2* | *Hap4* | *Hap1* | *Hap1* | *Hap1* | *Hap2* | *Hap1* | *Hap3* | *Hap1* | *Hap4* | ND | *Hap1* | *Hap2* | *Hap3* |
| Zhongdou39 ^III^ | *Hap1* | *Hap4* | *Hap2* | *Hap2* | *Hap1* | *Hap1* | *Hap1* | *Hap1* | ND | *Hap1* | *Hap3* | *Hap1* | *Hap3* | *Hap2* | *Hap1* | ND | *Hap1* | *Hap1* | *Hap1* |
| Xudou9 ^III^ | *Hap9* | ND | ND | *Hap1* | *Hap2* | *Hap1* | *Hap1* | *Hap1* | *Hap1* | *Hap1* | *Hap1* | *Hap2* | ND | *Hap1* | ND | ND | *Hap1* | *Hap2* | *Hap3* |
| Tiefeng31 ^III^ | ND | *Hap1* | *Hap2* | *Hap1* | ND | *Hap1* | *Hap1* | *Hap1* | *Hap2* | *Hap1* | *Hap1* | *Hap1* | *Hap1* | *Hap1* | *Hap1* | *Hap1* | ND | *Hap1* | *Hap1* |
| Jindou19 ^III^ | ND | *Hap1* | *Hap5* | *Hap1* | *Hap1* | *Hap1* | *Hap1* | *Hap1* | *Hap1* | *Hap1* | *Hap2* | *Hap2* | *Hap2* | *Hap1* | *Hap1* | *Hap1* | *Hap1* | *Hap1* | *Hap2* |
| Huachun6 ^III^ | ND | *Hap1* | *Hap1* | *Hap2* | *Hap1* | *Hap1* | *Hap1* | *Hap1* | *Hap1* | *Hap1* | *Hap2* | *Hap1* | *Hap3* | *Hap1* | *Hap1* | *Hap1* | *Hap2* | *Hap2* | *Hap3* |
| Huaidou9 ^III^ | ND | *Hap1* | *Hap2* | *Hap1* | *Hap1* | *Hap1* | *Hap1* | *Hap1* | *Hap3* | *Hap1* | *Hap5* | *Hap1* | *Hap3* | *Hap1* | *Hap1* | *Hap1* | *Hap1* | *Hap1* | *Hap1* |
| Flyer ^IV^ | *Hap2* | *Hap1* | *Hap1* | *Hap1* | *Hap1* | *Hap1* | *Hap1* | *Hap1* | *Hap1* | *Hap1* | *Hap1* | *Hap2* | *Hap1* | *Hap1* | *Hap1* | *Hap1* | *Hap2* | *Hap1* | *Hap1* |
| Omaha ^IV^ | *Hap2* | *Hap1* | *Hap1* | *Hap1* | *Hap1* | *Hap1* | *Hap2* | *Hap1* | *Hap1* | *Hap1* | *Hap1* | *Hap2* | *Hap6* | *Hap1* | *Hap1* | *Hap1* | *Hap2* | *Hap1* | *Hap3* |
| Calhoun ^IV^ | *Hap1* | *Hap1* | *Hap1* | *Hap1* | *Hap1* | *Hap1* | *Hap1* | *Hap1* | *Hap1* | *Hap1* | *Hap7* | *Hap1* | *Hap2* | *Hap1* | *Hap1* | *Hap1* | *Hap2* | *Hap1* | *Hap1* |
| CF461 ^IV^ | *Hap1* | *Hap1* | *Hap1* | *Hap1* | *Hap1* | *Hap1* | *Hap1* | *Hap1* | ND | *Hap1* | *Hap1* | *Hap2* | *Hap1* | *Hap1* | *Hap1* | *Hap1* | *Hap2* | *Hap1* | *Hap1* |
| UA-4805 ^IV^ | *Hap4* | *Hap1* | *Hap2* | *Hap1* | *Hap1* | *Hap1* | *Hap1* | *Hap1* | *Hap1* | *Hap1* | *Hap1* | *Hap1* | *Hap7* | *Hap1* | *Hap1* | *Hap1* | *Hap1* | *Hap1* | *Hap2* |
| Zheng92116 ^IV^ | *Hap11* | *Hap1* | *Hap2* | *Hap1* | *Hap2* | *Hap1* | *Hap1* | *Hap1* | *Hap1* | *Hap1* | *Hap6* | *Hap1* | *Hap3* | *Hap1* | *Hap1* | *Hap3* | *Hap1* | *Hap2* | *Hap1* |
| Guandou2 ^IV^ | *Hap8* | *Hap2* | *Hap2* | *Hap1* | *Hap1* | *Hap1* | *Hap1* | *Hap1* | *Hap1* | *Hap1* | *Hap6* | *Hap1* | *Hap2* | *Hap2* | *Hap1* | ND | *Hap1* | *Hap1* | *Hap2* |
| Jindou39 ^IV^ | ND | *Hap1* | *Hap2* | *Hap1* | *Hap2* | *Hap2* | *Hap1* | *Hap1* | *Hap1* | ND | *Hap1* | *Hap1* | *Hap3* | *Hap1* | *Hap1* | *Hap1* | *Hap3* | *Hap2* | *Hap1* |
| Shanning16 ^IV^ | ND | *Hap2* | *Hap2* | *Hap1* | *Hap1* | *Hap2* | *Hap1* | *Hap1* | *Hap1* | ND | *Hap1* | *Hap1* | *Hap2* | *Hap1* | *Hap1* | ND | *Hap1* | *Hap1* | *Hap1* |

**ND: Not available data**

**Table** **S3** Continued

| Variety Name | *GmCOL2* | *GmCOL4* | *GmCOL5* | *GmCOL6* | *GmCOL8* | *GmCOL9* | *GmCOL10* | *GmCOL13* | *GmCOL14* | *GmCOL15* | *GmCOL16* | *GmCOL19* | *GmCOL20* | *GmCOL22* | *GmCOL23* | *GmCOL24* | *GmCOL25* | *GmCOL26* | *GmCOL28* |
| --- | --- | --- | --- | --- | --- | --- | --- | --- | --- | --- | --- | --- | --- | --- | --- | --- | --- | --- | --- |
| Houzimao ^IV^ | *Hap1* | *Hap5* | *Hap2* | *Hap2* | *Hap1* | *Hap2* | *Hap1* | *Hap1* | *Hap1* | *Hap1* | *Hap6* | *Hap2* | *Hap3* | *Hap1* | *Hap1* | *Hap1* | *Hap1* | *Hap3* | *Hap2* |
| Nathan ^V^ | *Hap1* | *Hap1* | *Hap1* | *Hap1* | *Hap1* | *Hap1* | *Hap1* | *Hap1* | *Hap1* | *Hap1* | *Hap3* | *Hap1* | *Hap10* | *Hap1* | *Hap1* | ND | *Hap1* | *Hap1* | *Hap1* |
| Holladay ^V^ | *Hap3* | *Hap1* | *Hap1* | *Hap1* | *Hap1* | *Hap1* | *Hap2* | *Hap1* | *Hap1* | *Hap1* | *Hap2* | *Hap1* | *Hap1* | *Hap1* | *Hap1* | ND | *Hap1* | *Hap2* | *Hap2* |
| Hutcheson ^V^ | *Hap4* | *Hap1* | *Hap2* | *Hap1* | *Hap1* | *Hap1* | *Hap1* | *Hap1* | *Hap1* | *Hap1* | *Hap2* | *Hap1* | *Hap2* | *Hap1* | *Hap1* | *Hap1* | *Hap1* | *Hap1* | *Hap2* |
| R01-3474F ^V^ | *Hap4* | *Hap1* | *Hap2* | *Hap1* | ND | *Hap1* | *Hap1* | *Hap1* | *Hap1* | *Hap1* | *Hap2* | *Hap1* | *Hap1* | *Hap1* | *Hap1* | *Hap1* | *Hap1* | *Hap1* | *Hap2* |
| TN04-5321 ^V^ | *Hap2* | *Hap1* | *Hap2* | *Hap1* | *Hap1* | *Hap1* | *Hap1* | *Hap1* | *Hap1* | *Hap1* | *Hap3* | *Hap2* | *Hap4* | *Hap1* | *Hap1* | *Hap1* | *Hap1* | *Hap1* | ND |
| Shangdou14 ^V^ | ND | *Hap3* | *Hap2* | *Hap1* | *Hap2* | *Hap2* | *Hap1* | *Hap1* | *Hap1* | *Hap1* | *Hap1* | *Hap1* | *Hap4* | *Hap4* | *Hap1* | *Hap1* | *Hap4* | *Hap2* | *Hap3* |
| Dian86-4 ^V^ | ND | *Hap1* | *Hap2* | *Hap2* | *Hap1* | *Hap2* | *Hap1* | *Hap1* | *Hap1* | *Hap1* | *Hap1* | *Hap1* | *Hap1* | *Hap1* | *Hap1* | *Hap1* | *Hap1* | *Hap1* | *Hap1* |
| Diandou7 ^V^ | ND | *Hap1* | *Hap1* | *Hap2* | *Hap1* | *Hap2* | *Hap1* | *Hap1* | *Hap1* | *Hap1* | *Hap1* | *Hap1* | *Hap3* | *Hap1* | ND | *Hap1* | *Hap1* | *Hap2* | *Hap1* |
| Desha ^VI^ | *Hap4* | *Hap1* | *Hap2* | *Hap2* | *Hap1* | *Hap1* | *Hap1* | *Hap1* | *Hap1* | *Hap1* | *Hap2* | *Hap1* | *Hap2* | *Hap1* | *Hap1* | *Hap1* | *Hap1* | *Hap1* | *Hap1* |
| Musen ^VI^ | *Hap3* | *Hap1* | *Hap2* | *Hap1* | *Hap1* | *Hap1* | *Hap1* | *Hap1* | *Hap1* | *Hap1* | *Hap2* | *Hap3* | *Hap1* | *Hap1* | *Hap1* | *Hap1* | *Hap1* | *Hap1* | *Hap1* |
| D95-6271 ^VI^ | *Hap2* | *Hap1* | *Hap2* | *Hap1* | ND | *Hap1* | *Hap1* | *Hap1* | *Hap1* | *Hap1* | *Hap1* | *Hap4* | *Hap2* | *Hap1* | ND | *Hap1* | ND | *Hap1* | ND |
| G01-PR16 ^VI^ | *Hap2* | *Hap1* | *Hap2* | *Hap2* | *Hap1* | *Hap1* | *Hap2* | *Hap1* | *Hap1* | ND | *Hap3* | *Hap1* | *Hap2* | *Hap1* | *Hap1* | *Hap1* | *Hap1* | *Hap2* | ND |
| Boggs ^VI^ | *Hap2* | *Hap1* | ND | ND | ND | ND | ND | *Hap1* | *Hap1* | *Hap1* | *Hap3* | *Hap1* | *Hap2* | *Hap1* | ND | ND | ND | *Hap2* | ND |
| Zhongdou38 ^VI^ | ND | ND | *Hap2* | *Hap2* | *Hap2* | *Hap1* | ND | *Hap2* | *Hap1* | *Hap1* | *Hap3* | *Hap1* | ND | *Hap1* | ND | *Hap2* | *Hap1* | *Hap2* | *Hap1* |
| Wuhuasiyuehuang ^VI^ | ND | *Hap2* | *Hap2* | *Hap2* | *Hap2* | *Hap1* | *Hap1* | *Hap1* | *Hap1* | *Hap1* | *Hap4* | *Hap2* | *Hap3* | *Hap2* | *Hap1* | *Hap1* | *Hap3* | *Hap2* | *Hap3* |

**ND: Not available data**

**Table** **S3** Continued

| Variety Name | *GmCOL2* | *GmCOL4* | *GmCOL5* | *GmCOL6* | *GmCOL8* | *GmCOL9* | *GmCOL10* | *GmCOL13* | *GmCOL14* | *GmCOL15* | *GmCOL16* | *GmCOL19* | *GmCOL20* | *GmCOL22* | *GmCOL23* | *GmCOL24* | *GmCOL25* | *GmCOL26* | *GmCOL28* |
| --- | --- | --- | --- | --- | --- | --- | --- | --- | --- | --- | --- | --- | --- | --- | --- | --- | --- | --- | --- |
| Suxiandou19 ^VI^ | ND | *Hap1* | *Hap2* | *Hap1* | *Hap1* | *Hap2* | *Hap1* | *Hap1* | *Hap1* | ND | *Hap1* | *Hap1* | *Hap2* | *Hap1* | ND | ND | *Hap1* | *Hap2* | *Hap2* |
| NDnnong493/1 ^VI^ | *Hap2* | *Hap1* | *Hap4* | *Hap1* | *Hap2* | *Hap2* | *Hap2* | *Hap1* | *Hap1* | *Hap1* | *Hap1* | *Hap1* | *Hap4* | *Hap1* | ND | *Hap3* | ND | *Hap4* | *Hap1* |
| Stonewall ^VII^ | *Hap4* | *Hap1* | ND | *Hap1* | ND | ND | ND | *Hap1* | *Hap1* | *Hap1* | *Hap4* | *Hap1* | *Hap2* | *Hap1* | ND | ND | ND | *Hap1* | *Hap1* |
| Santee ^VII^ | *Hap2* | *Hap3* | *Hap2* | *Hap1* | *Hap1* | *Hap1* | *Hap1* | *Hap1* | *Hap1* | *Hap1* | *Hap3* | *Hap1* | ND | *Hap6* | *Hap1* | *Hap1* | *Hap1* | *Hap1* | *Hap1* |
| Hagood ^VII^ | *Hap2* | *Hap1* | ND | ND | ND | ND | *Hap1* | *Hap1* | *Hap1* | *Hap1* | *Hap3* | ND | *Hap2* | *Hap1* | ND | ND | *Hap1* | *Hap1* | *Hap1* |
| Benning ^VII^ | *Hap4* | *Hap1* | *Hap2* | *Hap1* | ND | *Hap1* | ND | *Hap1* | *Hap1* | ND | *Hap3* | *Hap1* | ND | *Hap1* | ND | *Hap1* | *Hap1* | *Hap1* | *Hap1* |
| Hunagfengwu ^VII^ | ND | *Hap2* | ND | *Hap2* | *Hap1* | *Hap1* | ND | *Hap1* | *Hap1* | *Hap1* | *Hap5* | *Hap1* | *Hap3* | *Hap1* | ND | *Hap1* | *Hap3* | *Hap1* | *Hap2* |
| Tongshanbopihuang ^VII^ | *Hap3* | *Hap4* | *Hap2* | *Hap2* | *Hap1* | *Hap2* | *Hap1* | *Hap3* | *Hap1* | *Hap1* | *Hap2* | *Hap1* | *Hap3* | *Hap1* | *Hap1* | *Hap1* | *Hap1* | *Hap2* | *Hap2* |
| Motte ^VIII^ | *Hap2* | *Hap1* | *Hap2* | *Hap1* | ND | ND | ND | *Hap1* | *Hap1* | *Hap1* | *Hap2* | *Hap1* | *Hap2* | *Hap1* | *Hap1* | *Hap1* | ND | *Hap1* | *Hap1* |
| Dowling ^VIII^ | *Hap2* | *Hap3* | *Hap2* | *Hap1* | *Hap1* | *Hap1* | *Hap1* | *Hap1* | *Hap1* | *Hap1* | *Hap3* | *Hap1* | *Hap2* | *Hap4* | *Hap1* | *Hap1* | *Hap1* | *Hap1* | *Hap1* |
| Crockett ^VIII^ | *Hap2* | *Hap1* | *Hap1* | *Hap2* | *Hap1* | *Hap2* | *Hap1* | *Hap1* | *Hap1* | *Hap1* | *Hap1* | *Hap1* | *Hap3* | *Hap1* | *Hap1* | *Hap1* | *Hap1* | *Hap2* | *Hap1* |
| Hengyangbayueqing ^VIII^ | *Hap3* | *Hap1* | *Hap2* | *Hap2* | *Hap2* | *Hap2* | *Hap1* | *Hap1* | ND | *Hap1* | *Hap1* | *Hap1* | *Hap2* | *Hap1* | *Hap1* | *Hap1* | *Hap1* | *Hap1* | *Hap2* |
| NDnxiadou25 ^VIII?^ | *Hap7* | *Hap1* | ND | *Hap2* | *Hap1* | *Hap2* | *Hap1* | *Hap1* | *Hap1* | *Hap1* | *Hap1* | *Hap1* | *Hap3* | *Hap1* | ND | ND | *Hap3* | *Hap1* | *Hap1* |
| Prichard ^VIII^ | *Hap2* | *Hap1* | ND | ND | ND | ND | *Hap1* | *Hap1* | *Hap1* | ND | *Hap9* | *Hap5* | *Hap2* | *Hap1* | *Hap1* | ND | ND | *Hap1* | *Hap2* |
| CIGRAS-51 ^VIII^ | *Hap2* | *Hap1* | ND | *Hap1* | ND | *Hap1* | *Hap1* | *Hap1* | ND | *Hap1* | *Hap2* | *Hap1* | *Hap2* | *Hap1* | *Hap1* | ND | *Hap1* | *Hap2* | *Hap1* |
| CIGRAS-06 ^VIII^ | *Hap1* | *Hap1* | *Hap2* | *Hap1* | *Hap1* | *Hap1* | *Hap1* | *Hap1* | ND | *Hap1* | *Hap1* | *Hap2* | *Hap1* | *Hap1* | *Hap1* | *Hap1* | *Hap1* | *Hap2* | *Hap1* |

**ND: Not available data**

**Table** **S3** Continued

| Variety Name | *GmCOL2* | *GmCOL4* | *GmCOL5* | *GmCOL6* | *GmCOL8* | *GmCOL9* | *GmCOL10* | *GmCOL13* | *GmCOL14* | *GmCOL15* | *GmCOL16* | *GmCOL19* | *GmCOL20* | *GmCOL22* | *GmCOL23* | *GmCOL24* | *GmCOL25* | *GmCOL26* | *GmCOL28* |
| --- | --- | --- | --- | --- | --- | --- | --- | --- | --- | --- | --- | --- | --- | --- | --- | --- | --- | --- | --- |
| Aijiaoqing ^VIII^ | ND | *Hap1* | *Hap2* | *Hap1* | *Hap1* | *Hap2* | *Hap1* | *Hap1* | *Hap1* | *Hap1* | *Hap2* | *Hap1* | ND | *Hap1* | ND | *Hap1* | *Hap1* | *Hap1* | *Hap2* |
| Pingguohuangdou ^VIII^ | *Hap6* | ND | *Hap2* | *Hap2* | *Hap1* | *Hap1* | *Hap1* | *Hap1* | *Hap1* | *Hap1* | *Hap1* | *Hap1* | *Hap1* | *Hap1* | *Hap1* | *Hap1* | *Hap4* | *Hap2* | *Hap1* |
| Nandou12 ^VIII^ | *Hap3* | *Hap2* | *Hap1* | *Hap2* | *Hap1* | *Hap5* | *Hap1* | *Hap2* | *Hap1* | ND | *Hap3* | *Hap1* | *Hap2* | *Hap1* | ND | *Hap1* | *Hap1* | *Hap1* | *Hap1* |
| Shangraodaqingsi ^VIII^ | *Hap3* | ND | *Hap2* | *Hap2* | *Hap1* | *Hap6* | *Hap1* | *Hap2* | *Hap1* | *Hap3* | *Hap2* | *Hap1* | ND | *Hap1* | *Hap1* | *Hap1* | *Hap2* | *Hap1* | *Hap2* |
| Zigongdongdou ^VIII^ | *Hap8* | *Hap1* | *Hap2* | *Hap2* | *Hap2* | *Hap1* | ND | *Hap1* | *Hap1* | *Hap3* | *Hap5* | *Hap1* | *Hap2* | *Hap2* | *Hap1* | ND | *Hap2* | *Hap1* | *Hap1* |
| Lanxidaqingdou ^VIII^ | *Hap7* | ND | *Hap1* | *Hap1* | ND | *Hap1* | *Hap1* | *Hap1* | *Hap1* | *Hap1* | *Hap2* | *Hap1* | ND | *Hap1* | ND | *Hap1* | *Hap1* | ND | *Hap2* |
| Qiudou 1 ^VIII^ | *Hap3* | *Hap1* | *Hap1* | *Hap2* | *Hap1* | *Hap1* | *Hap1* | *Hap1* | *Hap1* | *Hap4* | *Hap1* | *Hap1* | *Hap2* | *Hap1* | ND | *Hap1* | *Hap4* | *Hap2* | *Hap2* |
| Nandou17 ^VIII^ | *Hap7* | *Hap4* | *Hap1* | *Hap2* | *Hap1* | *Hap2* | ND | *Hap1* | *Hap1* | *Hap1* | *Hap5* | *Hap1* | ND | *Hap1* | ND | *Hap1* | *Hap1* | *Hap1* | ND |
| Jiangledaqingdou ^VIII^ | *Hap3* | ND | *Hap1* | *Hap2* | *Hap1* | *Hap2* | ND | *Hap2* | *Hap1* | *Hap1* | *Hap3* | *Hap2* | ND | *Hap1* | ND | *Hap1* | *Hap4* | *Hap2* | *Hap3* |
| Guixia3 ^VIII^ | *Hap3* | *Hap1* | *Hap2* | *Hap2* | ND | ND | *Hap1* | *Hap1* | *Hap1* | *Hap1* | *Hap3* | *Hap1* | *Hap2* | *Hap1* | ND | *Hap1* | *Hap4* | *Hap1* | *Hap2* |
| Jupiter ^IX^ | *Hap3* | *Hap1* | *Hap2* | *Hap1* | *Hap1* | *Hap2* | *Hap1* | *Hap1* | *Hap1* | *Hap1* | *Hap2* | *Hap2* | *Hap2* | *Hap1* | *Hap1* | *Hap1* | *Hap1* | *Hap1* | *Hap2* |
| Alamo ^IX^ | *Hap2* | *Hap1* | *Hap2* | *Hap1* | ND | ND | *Hap1* | *Hap1* | *Hap1* | ND | ND | *Hap1* | *Hap4* | *Hap2* | ND | *Hap1* | ND | *Hap2* | ND |
| FT-15 ^IX^ | *Hap3* | *Hap1* | *Hap1* | *Hap1* | *Hap1* | *Hap1* | *Hap2* | *Hap1* | *Hap1* | *Hap1* | *Hap2* | *Hap1* | *Hap2* | *Hap1* | *Hap1* | ND | *Hap1* | *Hap2* | *Hap2* |
| UFV-3 ^IX^ | ND | ND | *Hap1* | *Hap1* | *Hap1* | *Hap1* | *Hap1* | *Hap1* | *Hap1* | ND | *Hap3* | *Hap1* | *Hap2* | *Hap1* | *Hap1* | *Hap2* | *Hap3* | *Hap2* | *Hap2* |
| IAC-8 ^IX^ | *Hap2* | *Hap1* | *Hap1* | *Hap1* | ND | ND | ND | *Hap1* | *Hap1* | *Hap1* | *Hap2* | *Hap2* | *Hap2* | *Hap1* | *Hap1* | *Hap1* | *Hap1* | *Hap4* | *Hap1* |
| I.C.-192 ^X^ | *Hap3* | ND | *Hap1* | *Hap2* | *Hap1* | *Hap1* | *Hap2* | *Hap1* | *Hap1* | *Hap1* | *Hap6* | *Hap1* | *Hap4* | *Hap4* | *Hap2* | *Hap1* | *Hap1* | *Hap1* | *Hap2* |

**ND: Not available data**
