# Supplementary material for: CONSTANS Polymorphism Modulates Flowering Time and Maturity in Soybean
Source: Front Plant Sci. 2022 Mar 17;13:817544. doi: 10.3389/fpls.2022.817544 (PMC8969907; doi:10.3389/fpls.2022.817544)
Supplement: Supplementary file 6 [file Table_4.docx]

**Table S4** Polymorphic sites of 21 soybean haplotypes

| **Gene** | **No. of Sites used for haplotypes** | **Types of sites** | **No. of Sites used for tagging haplotypes** |
| --- | --- | --- | --- |
| *GmCOL2* | 46 | 34 SNPs, 12 InDels | 7 |
| *GmCOL4* | 121 | 83 SNPs, 38 InDels | 5 |
| *GmCOL5* | 433 | 22 SNPs, 411 InDels | 4 |
| *GmCOL6* | 114 | 22SNPs, 92 InDels | 7 |
| *GmCOL8* | 5 | 5 SNPs | 2 |
| *GmCOL9* | 30 | 18 SNPs, 12 InDels | 6 |
| *GmCOL10* | 10 | 4 SNPs, 6 InDels | 3 |
| *GmCOL13* | 4 | 4 SNPs | 4 |
| *GmCOL14* | 19 | 3 SNPs, 16 InDels | 3 |
| *GmCOL15* | 3 | 3SNPs | 3 |
| *GmCOL16* | 45 | 29 SNPs, 16 InDels | 8 |
| *GmCOL19* | 7 | 7 SNPs | 4 |
| *GmCOL20* | 41 | 25 SNPs, 16 InDels | 7 |
| *GmCOL22* | 51 | 25 SNPs, 26 InDels | 10 |
| *GmCOL23* | 32 | 18 SNPs, 14 InDels | 7 |
| *GmCOL24* | 24 | 19 SNPs, 5 InDels | 7 |
| *GmCOL25* | 32 | 21 SNPs, 11 InDels | 10 |
| *GmCOL26* | 24 | 24 SNPs | 16 |
| *GmCOL28* | 38 | 23 SNPs, 15 InDels | 2 |
