# Supplementary material for: CONSTANS Polymorphism Modulates Flowering Time and Maturity in Soybean
Source: Front Plant Sci. 2022 Mar 17;13:817544. doi: 10.3389/fpls.2022.817544 (PMC8969907; doi:10.3389/fpls.2022.817544)
Supplement: Supplementary file 7 [file Table_5.docx]

**Table S5** Types of different soybean *GmCOL* proteins and their haplotype distribution

| Gene | Types of protein | Amino acids in mutation sites | Haplotype distribution |
| --- | --- | --- | --- |
| *GmCOL2* | P0 |  | *Hap1, Hap5, Hap4, Hap10* |
|  | P1 | D_216_ | *Hap2, Hap3, Hap6, Hap11* |
|  | P2 | I_116_ | *Hap8* |
|  | P3 | I_116_ L_176_ | *Hap9* |
|  | P4 | A_103_ N_104_ D_217_ | *Hap7* |
| *GmCOL4* | P0 |  | *Hap1, Hap2, Hap3, Hap4* |
|  | P1 | R_104_ | *Hap5* |
| *GmCOL5* | P0 |  | *Hap1* |
|  | P1 | E_167_ | *Hap2, Hap4, Hap5* |
|  | P2 | E_167_ L_265_ | *Hap3* |
| *GmCOL6* | P0 |  | *Hap1, Hap2* |
|  | P1 | *Del_153_ | *Hap1, Hap2* |
|  | P 2 | *Del_153_ *Del_166-185_ L_220_ R_295_ | *Hap3, Hap5* |
|  | P3 | *Del_79_ *Del_153_ | *Hap1* |
|  | P4 | L_220_ P_323_ Q_404_ | *Hap4* |
|  | P5 | P^323^ | *Hap2* |
|  | P6 | P_98_ L_99_ Stop codon_104_ | *Hap1* |
| *GmCOL8* | P0 |  | *Hap1, Hap2* |
| *GmCOL9* | P0 |  | *Hap1, Hap2, Hap3, Hap4, Hap5, Hap6, Hap7, Hap8, Hap9, Hap10, Hap11* |
| *GmCOL10* | P0 |  | *Hap1* |
|  | P1 | S_364_ | *Hap2, Hap3, Hap4* |
| *GmCOL13* | P0 |  | *Hap1, Hap2, Hap3* |

**Table S5** Continued

| Gene | Types of protein | Amino acids in mutation sites | Haplotype distribution |
| --- | --- | --- | --- |
| *GmCOL14* | P0 |  | *Hap1* |
|  | P1 | Stop codon_59_ | *Hap1* |
|  | P2 | T_66_ | *Hap2* |
|  | P3 | Stop codon_110_ | *Hap1* |
|  | P4 | *Del_291_ | *Hap1* |
|  | P5 | P_292_ | *Hap3* |
| *GmCOL15* | P0 |  | *Hap1, Hap2, Hap3* |
|  | P1 | Q_328_ | *Hap4* |
| *GmCOL16* | P0 |  | *Hap1, Hap2, Hap4, Hap6, Hap9* |
|  | P1 | A_101_ | *Hap3, Hap5, Hap7, Hap8* |
| *GmCOL19* | P0 |  | *Hap1, Hap5* |
|  | P1 | D_102_ | *Hap2, Hap3, Hap4* |
| *GmCOL20* | P0 |  | *Hap1, Hap6, Hap7, Hap8* |
|  | P1 | E_161_ | *Hap2, Hap3, Hap9* |
|  | P2 | Q_319_ | *Hap4, Hap5* |
|  | P3 | E_161_ | *Hap10* |
|  | P4 | V_103_ E_161_ Q_319_ | *Hap2* |
|  | P5 | Y_276_ Q_319_ | *Hap4* |
| *GmCOL22* | P0 |  | *Hap1, Hap4, Hap5, Hap6* |
|  | P1 | *Del_76_ | *Hap2* |
|  | P2 | A_274_ | *Hap3* |
|  | P3 | Stop codon_155_ | *Hap1, Hap4* |
|  | P4 | I_157_ | *Hap1* |
|  | P5 | Stop codon_229_ | *Hap1* |

Table S5 Continued

| **Gene** | **Types of protein** | **Amino acids in mutation sites** | **Haplotype distribution** |
| --- | --- | --- | --- |
| *GmCOL22* | P6 | Stop codon_229_ | *Hap1* |
|  | P7 | *Del_79-80_ | *Hap4* |
| *GmCOL23* | P0 |  | *Hap1* |
|  | P1 | Q_104_ Q_105_ Y_109_ | *Hap2, Hap3, Hap5* |
|  | P2 | Y_107_ G_352_ | *Hap6, Hap9* |
|  | P3 | T_104_ Y_108_ | *Hap8* |
|  | P4 | Y_107_ G_352_*Del_418_ | *Hap4* |
|  | P5 | Y_107_ Stop codon_434_ | *Hap10* |
|  | P6 | *Del_418_ | *Hap1* |
|  | P7 | Y_107_ | *Hap3* |
|  | P8 | K_431_ Stop codon_434_ | *Hap7* |
|  | P9 | G_354_ | *Hap4* |
| *GmCOL24* | P0 |  | *Hap1* |
|  | P1 | D_75_ M_92_ P_93_ R_112_ F_174_ | *Hap2, Hap3* |
|  | P2 | D_75_ M_92_ P_93_ R_112_ | *Hap5* |
|  | P3 | F_174_ | *Hap4* |
| *GmCOL25* | P0 |  | *Hap1, Hap4* |
|  | P1 | L_73_ I_84_ Y_108_ Stop codon_152_ | *Hap2, Hap5* |
|  | P2 | I_165_ | *Hap3* |
| *GmCOL26* | P0 |  | *Hap1* |
|  | P1 | A_8_ H_42_ K_55_ F_83_ K_95_ A_129_ | *Hap2* |
|  | P2 | A_8_ T_25_ M_60_ E_127_ A_129_ | *Hap3* |
|  | P3 | A_8_ T_39_ C_42_ I_59_ A_129_ | *Hap4* |

Table S5 Continued

| **Gene** | **Types of protein** | **Amino acids in mutation sites** | **Haplotype distribution** |
| --- | --- | --- | --- |
| *GmCOL28* | P0 |  | *Hap1* |
|  | P1 | A_88_ | *Hap2* |
|  | P2 | S_4_ | *Hap3* |

Note: 1. A_103_, subscript 103 means which position of soybean *GmCOL* protein sequence this amino acid is located in; others are the same as A_103_

2. *Del_153_ denotes deletion of nucleotide from the position of 153 bp of amino acid sites, others are the same as Del_153_
