# Supplementary material for: CONSTANS Polymorphism Modulates Flowering Time and Maturity in Soybean
Source: Front Plant Sci. 2022 Mar 17;13:817544. doi: 10.3389/fpls.2022.817544 (PMC8969907; doi:10.3389/fpls.2022.817544)
Supplement: Supplementary file 8 [file Table_6.docx]

**Table S6** ANOVA for Haplotype of *GmCOL* gene family

***GmCOL2*: Summary of 2015**

| Group | Count | Sum | Average | Variance |
| --- | --- | --- | --- | --- |
| *Hap1* | 18 | 670.9 | 37.27 | 283.01 |
| *Hap2* | 40 | 1781.6 | 44.54 | 605.49 |
| *Hap3* | 19 | 1282.4 | 67.49 | 1319.78 |
| *Hap4* | 16 | 640.3 | 40.02 | 382.44 |

| Source of variation | DF | SS | MS | F | P |
| --- | --- | --- | --- | --- | --- |
| Between Groups | 3 | 10705.66 | 3568.55 | 5.48 | **0.002** |
| Within Groups | 89 | 57917.98 | 650.764 |  |  |
| Total | 92 | 68623.65 |  |  |  |

| Source of variation | DF | SS | MS | F | P |
| --- | --- | --- | --- | --- | --- |
| Between Groups | 3 | 11021.16857 | 3673.72 | 4.87 | **0.003** |
| Within Groups | 91 | 68579.53856 | 753.62 |  |  |
| Total | 94 | 79600.70 |  |  |  |

| Group | Count | Sum | Average | Variance |
| --- | --- | --- | --- | --- |
| *Hap1* | 18 | 606.23 | 33.68 | 252.87 |
| *Hap2* | 40 | 1840.15 | 46.00 | 887.35 |
| *Hap3* | 20 | 1316.1 | 65.81 | 1106.39 |
| *Hap4* | 17 | 688.59 | 40.51 | 540.81 |

***GmCOL2*: Summary of 2016**

| Source of variation | DF | SS | MS | F | P |
| --- | --- | --- | --- | --- | --- |
| Between Groups | 1 | 4216.99 | 4216.99 | 5.48 | **0.021** |
| Within Groups | 102 | 78410.78 | 768.73 |  |  |
| Total | 103 | 82627.77 |  |  |  |

| Group | Count | Sum | Average | Variance |
| --- | --- | --- | --- | --- |
| *Hap1* | 50 | 2172.2 | 43.44 | 791.61 |
| *Hap2* | 54 | 3034.2 | 56.19 | 747.58 |

***GmCOL5*: Summary of 2015**

| Source of variation | DF | SS | MS | F | P |
| --- | --- | --- | --- | --- | --- |
| Between Groups | 1 | 5374.67 | 5374.67 | 6.81 | **0.01039** |
| Within Groups | 104 | 82070.36 | 789.1381 |  |  |
| Total | 105 | 87445.03 |  |  |  |

| Group | Count | Sum | Average | Variance |
| --- | --- | --- | --- | --- |
| *Hap1* | 50 | 2023.41 | 40.47 | 760.53 |
| *Hap2* | 56 | 3065.02 | 54.73 | 814.63 |

***GmCOL5*: Summary of 2016**

**Table S6** Continued

| Group | Count | Sum | Average | Variance |
| --- | --- | --- | --- | --- |
| *Hap1* | 67 | 3414.8 | 50.97 | 678.63 |
| *Hap2* | 24 | 1430.6 | 59.61 | 924.12 |
| *Hap3* | 4 | 93.9 | 23.47 | 1.12 |
| *Hap4* | 4 | 91.8 | 22.95 | 1.74 |

***GmCOL9*: Summary of 2015**

| Source of variation | DF | SS | MS | F | P |
| --- | --- | --- | --- | --- | --- |
| Between Groups | 3 | 7953.03 | 2651.01 | 3.81277 | **0.012528** |
| Within Groups | 95 | 66053.28 | 695.2977 |  |  |
| Total | 98 | 74006.31 |  |  |  |

| Group | Count | Sum | Average | Variance |
| --- | --- | --- | --- | --- |
| *Hap1* | 67 | 3252.71 | 48.55 | 679.19 |
| *Hap2* | 24 | 1353.46 | 56.39 | 1067.77 |
| *Hap3* | 4 | 73.93 | 18.48 | 13.083 |
| *Hap4* | 4 | 95.7 | 23.925 | 1.21 |

***GmCOL9*: Summary of 2016**

| Source of variation | DF | SS | MS | F | P |
| --- | --- | --- | --- | --- | --- |
| Between Groups | 3 | 7509.03 | 2503.01 | 3.42 | **0.0203** |
| Within Groups | 95 | 69428.05 | 730.82 |  |  |
| Total | 98 | 76937.08 |  |  |  |

| Group | Count | Sum | Average | Variance |
| --- | --- | --- | --- | --- |
| *Hap1* | 119 | 5759.1 | 48.39 | 740.53 |
| *Hap2* | 5 | 386.1 | 77.22 | 796.34 |
| *Hap3* | 1 | 86.7 | 86.7 |  |

***GmCOL13*: Summary of 2015**

| Source of variation | DF | SS | MS | F | P |
| --- | --- | --- | --- | --- | --- |
| Between Groups | 2 | 5355.15 | 2677.57 | 3.61 | 0.030 |
| Within Groups | 122 | 90568.54 | 742.36 |  |  |
| Total | 124 | 95923.67 |  |  |  |

| Group | Count | Sum | Average | Variance |
| --- | --- | --- | --- | --- |
| *Hap1* | 121 | 5662.43 | 46.80 | 809.29 |
| *Hap2* | 5 | 391.6 | 78.32 | 940.497 |
| *Hap3* | 1 | 93.2 | 93.2 |  |

| Source of variation | DF | SS | MS | F | P |
| --- | --- | --- | --- | --- | --- |
| Between Groups | 2 | 6794.01 | 3397.01 | 4.17 | 0.017 |
| Within Groups | 124 | 100876.78 | 813.52 |  |  |
| Total | 126 | 107670.8 |  |  |  |

***GmCOL13*: Summary of 2016**

**Table S6** Continued

***GmCOL15*: Summary of 2015**

| Group | Count | Sum | Average | Variance |
| --- | --- | --- | --- | --- |
| *Hap1* | 104 | 4994.5 | 48.02 | 663.43 |
| *Hap2* | 5 | 114.8 | 22.96 | 1.67 |
| *Hap3* | 2 | 219 | 109.5 | 264.5 |
| *Hap4* | 1 | 107 | 107 |  |

| Source of variation | DF | SS | MS | F | P |
| --- | --- | --- | --- | --- | --- |
| Between Groups | 3 | 14149.18 | 4716.39 | 7.42 | 0.0001 |
| Within Groups | 108 | 68604.42 | 635.23 |  |  |
| Total | 111 | 82753.59 |  |  |  |

***GmCOL15*: Summary of 2016**

| Group | Count | Sum | Average | Variance |
| --- | --- | --- | --- | --- |
| *Hap1* | 106 | 4963.1 | 46.82 | 792.82 |
| *Hap2* | 5 | 133.3 | 26.66 | 12.408 |
| *Hap3* | 2 | 166.2 | 83.1 | 188.18 |
| *Hap4* | 1 | 94.3 | 94.3 |  |

| Source of variation | DF | SS | MS | F | P |
| --- | --- | --- | --- | --- | --- |
| Between Groups | 3 | 6915.63 | 2305.22 | 3.04 | 0.032 |
| Within Groups | 110 | 83484.20 | 758.95 |  |  |
| Total | 113 | 90399.84 |  |  |  |

| Group | Count | Sum | Average | Variance |
| --- | --- | --- | --- | --- |
| *Hap1* | 52 | 2112.9 | 40.63 | 563.09 |
| *Hap2* | 25 | 1487 | 59.48 | 824.66 |
| *Hap3* | 23 | 1254.7 | 54.55 | 653.90 |
| *Hap4* | 6 | 251.2 | 41.87 | 676.70 |
| *Hap5* | 5 | 382.8 | 76.56 | 1581.77 |

***GmCOL16*: Summary of 2015**

| Source of variation | DF | SS | MS | F | P |
| --- | --- | --- | --- | --- | --- |
| Between Groups | 4 | 11176.30 | 2794.07 | 4.08 | 0.0041 |
| Within Groups | 106 | 72605.94 | 684.96 |  |  |
| Total | 110 | 83782.2364 |  |  |  |

***GmCOL16*: Summary of 2016**

| Group | Count | Sum | Average | Variance |
| --- | --- | --- | --- | --- |
| *Hap1* | 46 | 1443.02 | 31.37 | 192.32 |
| *Hap2* | 20 | 1004.4 | 50.22 | 693.83 |
| *Hap3* | 21 | 1119.06 | 53.288 | 983.61 |
| *Hap4* | 6 | 257.96 | 42.99 | 1021.04 |
| *Hap5* | 5 | 310.33 | 62.07 | 1193.42 |

| Source of variation | DF | SS | MS | F | P |
| --- | --- | --- | --- | --- | --- |
| Between Groups | 4 | 11240.84 | 2810.21 | 5.085 | 0.0009 |
| Within Groups | 93 | 51388.33 | 552.56 |  |  |
| Total | 97 | 62629.17 |  |  |  |

**Table S6** Continued

***GmCOL25:* Summary of 2015**

| Group | Count | Sum | Average | Variance |
| --- | --- | --- | --- | --- |
| *Hap1* | 87 | 4023.2 | 46.24 | 665.07 |
| *Hap2* | 9 | 500.3 | 55.59 | 999.16 |
| *Hap3* | 6 | 439.8 | 73.3 | 577.01 |
| *Hap4* | 5 | 404.2 | 80.84 | 1035.95 |
| *Hap5* | 1 | 24.4 | 24.4 |  |

| Source of variation | DF | SS | MS | F | P |
| --- | --- | --- | --- | --- | --- |
| Between Groups | 4 | 10176.31 | 2544.08 | 3.62 | 0.008 |
| Within Groups | 103 | 72218.43 | 701.15 |  |  |
| Total | 107 | 82394.74 |  |  |  |

| Group | Count | Sum | Average | Variance |
| --- | --- | --- | --- | --- |
| *Hap1* | 88 | 3951.5 | 44.90 | 750.46 |
| *Hap2* | 9 | 403 | 44.78 | 511.33 |
| *Hap3* | 6 | 435.4 | 72.57 | 939.13 |
| *Hap4* | 6 | 465 | 77.5 | 1180.75 |
| *Hap5* | 1 | 25.3 | 25.3 |  |

| Source of variation | DF | SS | MS | F | P |
| --- | --- | --- | --- | --- | --- |
| Between Groups | 4 | 10295.18 | 2573.79 | 3.38 | 0.012 |
| Within Groups | 105 | 79980.52 | 761.72 |  |  |
| Total | 109 | 90275.71 |  |  |  |

***GmCOL25*: Summary of 2016**

| Group | Count | Sum | Average | Variance |
| --- | --- | --- | --- | --- |
| *Hap1* | 58 | 2814.6 | 48.53 | 577.82 |
| *Hap2* | 27 | 1841.6 | 68.21 | 986.82 |
| *Hap3* | 22 | 789.5 | 35.89 | 399.63 |

| Source of variation | DF | SS | MS | F | P |
| --- | --- | --- | --- | --- | --- |
| Between Groups | 2 | 13373.20 | 6686.60 | 10.38 | 7.7495E-05 |
| Within Groups | 104 | 66985.18 | 644.09 |  |  |
| Total | 106 | 80358.38 |  |  |  |

***GmCOL28*: Summary of 2015**

| Group | Count | Sum | Average | Variance |
| --- | --- | --- | --- | --- |
| *Hap1* | 58 | 2807.1 | 48.40 | 712.27 |
| *Hap2* | 29 | 1846.58 | 63.67 | 1076.43 |
| *Hap3* | 22 | 751.43 | 34.15 | 408.67 |

| Source of variation | DF | SS | MS | F | P |
| --- | --- | --- | --- | --- | --- |
| Between Groups | 2 | 11076.39 | 5538.19 | 7.40 | 0.00098 |
| Within Groups | 106 | 79321.71 | 748.32 |  |  |
| Total | 108 | 90398.11 |  |  |  |

***GmCOL28*: Summary of 2016**
